# CircPAK1 promotes the progression of hepatocellular carcinoma via modulation of YAP nucleus localization by interacting with 14-3-3ζ

**DOI:** 10.1186/s13046-022-02494-z

**Published:** 2022-09-22

**Authors:** Xiaopei Hao, Yao Zhang, Xiaoli Shi, Hanyuan Liu, Zhiying Zheng, Guoyong Han, Dawei Rong, Chuanyong Zhang, Weiwei Tang, Xuehao Wang

**Affiliations:** 1 Hepatobiliary/Liver Transplantation Center, The First Affiliated Hospital of Nanjing Medical University, Key Laboratory of Living Donor Transplantation, Chinese Academy of Medical Sciences, Nanjing, China; 2 School of Medicine, Southeast University, Nanjing, China; 3 Department of General Surgery, Nanjing First Hospital, Nanjing Medical University, Nanjing, China; 4 Department of Anesthesiology, The First Affiliated Hospital of Nanjing Medical University, Nanjing, China

**Keywords:** Hepatocellular carcinoma, circPAK1, CS/si-circPAK1 nanocomplexes, Hippo signaling pathway, Exosome, Lenvatinib resistance

## Abstract

**Background:**

Circular RNA (circRNA), a new class of non-coding RNA, has obvious correlations with the occurrence and development of many diseases, including tumors. This study aimed to investigate the potential roles of circPAK1 in hepatocellular carcinoma (HCC).

**Methods:**

High-throughput sequencing was performed on 3 pairs of HCC and matched normal tissues to determine the upregulated circRNAs. The expression level of circPAK1 was detected by qRT-PCR in HCC and paired with normal liver tissue samples. The effects of circPAK1 on proliferation, invasion, metastasis and apoptosis of HCC cells were evaluated by in vitro and in vivo experiments. We also constructed Chitosan/si-circPAK1 (CS/si-circPAK1) nanocomplexes using Chitosan material to evaluate its in vivo therapeutic effect on HCC. High-throughput sequencing, RNA-sequencing, RNA probe pull-down, RNA immunoprecipitation and Co-Immunoprecipitation assays were performed to explore the relationship between circPAK1, 14–3-3ζ, p-LATS1 and YAP. Exosomes isolated from lenvatinib-resistant HCC cell lines were used to evaluate the relationship between exosomal circPAK1 and lenvatinib resistance.

**Results:**

CircPAK1, a novel circRNA, is highly expressed in HCC tumor tissues and cell lines as well as correlated with poor outcomes in HCC patients. Functionally, circPAK1 knockdown inhibited HCC cell proliferation, migration, invasion and angiogenesis while circPAK1 overexpression promoted HCC progression. The tumor-promoting phenotypes of circPAK1 on HCC were also confirmed by animal experiments. Importantly, the application of CS/si-circPAK1 nanocomplexes showed a better therapeutic effect on tumor growth and metastasis. Mechanistically, circPAK1 enhanced HCC progression by inactivating the Hippo signaling pathway, and this kind of inactivation is based on its competitively binding of 14–3-3 ζ with YAP, which weakens the recruitment and cytoplasmic fixation of 14–3-3 ζ to YAP, thus promoting YAP nucleus localization. Additionally, circPAK1 could be transported by exosomes from lenvatinib-resistant cells to sensitive cells and induce lenvatinib resistance of receipt cells.

**Conclusion:**

CircPAK1 exerts its oncogenic function by competitively binding 14–3-3 ζ with YAP, thus promoting YAP nucleus localization, leading to the inactivation of a Hippo signaling pathway. Exosomal circPAK1 may drive resistance to lenvatinib, providing a potential therapeutic target for HCC patients.

**Supplementary Information:**

The online version contains supplementary material available at 10.1186/s13046-022-02494-z.

## Background

The incidence and mortality of Hepatocellular carcinoma (HCC) have been continuously rising in recent decades [1]. The treatment of HCC is a comprehensive treatment mode based on radical resection，still lacks effective molecular therapeutic targets. Though great efforts have been made to improve treatment options, HCC is still associated with a poor prognosis [1]. Therefore, research on the molecular mechanisms of HCC pathogenesis is urgently needed.

Circular RNAs (circRNAs) are members of newly discovered non-coding RNAs, initially disregarded as functionless products of pre-mRNA splicing errors [2]. However, an increasing number of circRNAs have been identified and studied, and their roles in organisms have been gradually discovered and considered pervasive regulatory molecules. Numerous studies have pointed out that circRNA is closely correlated with the pathogenesis of tumors, including HCC [3–6]. The classical research mechanisms related to circRNA include molecular sponge, m6A modification, RNA binding protein, translation, etc. [7–11]. In addition, circRNA can also be transported through extracellular vesicles, which has the potential to affect the chemosensitivity of tumor cells and function as targets of chemotherapy drugs for treating malignancies [12–14]. Moreover, the circRNA-based vaccine has been successfully designed and the vaccine can be rapidly generated by in vitro transcription without nucleotide modification, and has strong stability [10]. All the above-mentioned indicate that circRNA has extensive research value. However, a large number of circRNAs remain to be discovered and studied.

In the present study, we performed high-throughput sequencing on HCC and paired normal liver tissues and identified a significantly upregulated circRNA-a newly discovered circRNA--circPAK1, for the subsequent molecular mechanism exploration. Our data demonstrated that circPAK1 was significantly upregulated in HCC tissue and cell lines, and high levels of circPAK1 develop poor outcomes in HCC patients. We further validated the oncogenic role of circPAK1 through in vitro and in vivo experiments. Moreover, we constructed Chitosan /si-circPAK1 (CS/si-circPAK1) nanocomplexes and its effective inhibition of tumor growth and metastasis brought us new insight into molecular therapy combined with nanometer materials. Mechanistically, we demonstrated that circPAK1 functioned as an oncogene via directly binding to 14–3-3ζ to facilitate YAP nucleus translocation and inactivate Hippo signaling pathway. Importantly, we surprisingly found that circPAK1 can be transported by exosomes through lenvatinib-resistant cells to lenvatinib sensitive cells to decrease the sensitivity of HCC cells to lenvatinib. Taken together, circPAK1 can be taken as a promising therapeutic target in HCC treatment.

## Methods

### Patient samples

Overall, HCC tissue samples (60 cases) were collected from HCC patients who received a primary surgery attempt between September 2015 and August 2019 at the First Affiliated Hospital of Nanjing Medical University. Patients who lacked detailed clinical pathological information and who received preoperative chemotherapy or radiotherapy were excluded. Written informed consent was obtained from all patients and the study protocol was approved by the Ethical Committee of the First Affiliated Hospital of Nanjing Medical University.

### Cell lines and culture conditions

Human HCC cell lines, including Hep-G2, Focus, Hep-3B, MHCC-97 L, HCC-LM3 (LM3), YY8103, Huh-7, and L02 cell lines were purchased from the Chinese Academy of Sciences Cell Bank (CASCB, Shanghai, China). All the HCC cell lines were cultured with DMEM (Gibco, MD, USA). HUVECs were obtained from the American Type Culture Collection (ATCC, VA, USA) and cultured with RPMI-1640 (Gibco, MD, USA). All the cells were supplemented with 10% fetal bovine serum (HyClone, UT, USA) and 1% penicillin/streptomycin at 37 °C with 5% CO_2_ in an incubator.

### Cell line establishing and generation of lenvatinib-resistant cells

The shRNA targeting the junction site or random sequence of human circPAK1 was designed and synthesized to establish the sh-circPAK1 vector, while the lentiviral vectors containing human circPAK1 were also to construct the Lv-circPAK1 vector. The above lentiviral vectors were synthesized by GeneChem (Shanghai, China). After the transfection of lentiviral vectors into target cells, stably transduced cells were selected by puromycin and validated by qRT-PCR. The silencing RNA against YAP (si-YAP) and 14–3-3ζ (si-14-3-3ζ) were synthesized and purchased from Corues Bio (Nanjing, China). The target sequence of shRNA or siRNAs were listed in Table S1, and the full sequence of circPAK1 was listed in Table S2. We selected LM3 and Hep-3B cells to induce lenvatinib-resistant HCC cells. Lenvatinib was purchased from MCE (MedChemExpress, NJ, USA). Briefly, cells were exposed to graded drug concentrations (5 μmol/L to 25 μmol/L) step by step to induce lenvatinib resistance. After 4 months of induction, the two lenvatinib-resistant HCC cell lines were generated.

### Preparation of CS/si-circPAK1 nanocomplexes

CS/si-circPAK1 nanocomplexes was designed and synthesized by Shennuoqing Biotechnology (Nanjing, China).

### In vivo nude mouse model

A total of 84 female BALB/c nude mice (4–6 weeks old) were purchased from the Model Animal Research Center of Nanjing Medical University (NJMU). The mice were randomly divided into 12 groups of 6 each. All mice were housed in a pathogen-free environment, and animal experiments were authorized by the NJMU Institutional Animal Care and Use Committee.

To evaluate the roles circPAK1 played in vivo, we first randomly chose four groups to establish a subcutaneous tumor model: Two groups of mice were subcutaneously injected with 1 × 10^6^ LM3 cells infected with sh-circPAK1 or sh-NC, the other two groups subcutaneously injected with 1 × 10^6^ Hep-3B cells transfected with Lv-circPAK1 or Lv-NC. Tumor diameters were recorded every 4 days using a caliper. The tumor volume was calculated using the following formula: volume (mm^3^) = 0.5 × width^2^ × length. After 32 days, the mice were sacrificed to harvest and weigh the grafted tumors. To evaluate the roles of circPAK1 on lung metastasis, we set another 4 groups to receive tail vein injection transfected cells (LM3 and Hep-3B) to establish a lung metastasis model. The mice were sacrificed after 30 days, and the lungs were separated and stained with hematoxylin and eosin (H&E).

In addition, CS/si-circPAK1 nanocomplexes treatment and non-treatment groups were established. LM3 cells were used to generate subcutaneous xenograft tumor and lung metastasis models according to the above protocol. After 8 days, saline, si-circPAK1 (10 nmol, in vivo-grade cholesterol-conjugated RIG-I siRNA, RiboBio), or CS/si-circPAK1 nanocomplexes (10 nmol) were injected intratumorally (50 μl) or intravenously (200 μl) into the six groups, once every 4 days, respectively. Mice were treated as described above.

### Haematoxylin and eosin (H&E) and immunohistochemical (IHC) staining

Mice lung tissues were embedded in Paraffin and cut into 5 μm sections for H&E staining. While mice tumor tissues were fixed with 4% paraformaldehyde, dehydrated using ethanol solution, and then embedded in paraffinand cut into 4 μm sections, incubated with 3% H_2_O_2_ solution. Twenty minutes later, blocking the slice with blocking solution for 30 min, and incubating with the corresponding primary antibody at 4 °C overnight. The rest steps were consistent with previously described. Images were captured using a microscope (Leica Microsystems, Germany). The staining results were evaluated in a double-blind manner.

### RNA extraction and quantitative real-time polymerase chain reaction (qRT-PCR)

Total RNA was extracted from cells or frozen human tissues using RNA Quick Purification kit (YiShanbio, Shanghai, China) in accordance with the manufacturer’s instructions. Reverse transcription was performed using a cDNA kit (Vazyme Biotech, Nanjing, China) to synthesize the cDNA. All the primer sequences used in this study were listed in Table S3. qRT-PCR was performed using SYBR Green PCR kits (Vazyme Biotech, Nanjing, China) on the ABI 7900 detection system (Applied Biosystems, CA, USA). and the CT values were determined. The relative expression of circPAK1 and mRNA were calculated using the 2^-ΔΔCt^ method. GAPDH and U6 were used as internal standards.

### Western blot

Proteins were extracted by using RIPA buffer (Beyotime, Shanghai, China) containing protease inhibitors. Protein lysates were separated using 10% sodium dodecyl sulfate-polyacrylamide gels (SDS-PAGE) and subsequently transferred onto polyvinylidene fluoride membranes (Millipore, MA, USA). The membranes were subsequently blocked with fast blocking buffer (Beyotime, Shanghai, China) for 50 min and then incubated with primary antibodies at 4 °C overnight. After incubating with the respective secondary antibodies for 1 h and washing three times every 10 min with Tris-buffered saline with Tween-20 (TBST), the ECL signals were visualised by using ECL Western Blotting Kit (Millipore, MA, USA). Antibodies used in this study were listed in Table S4.

### CCK-8 cell proliferation assay

Cell Counting Kit-8 was performed as we described before [15].

### Colony formation assay

For colony formation assay, cells were plated at a density of 600 cells per well into 6-well plates and cultured in complete media for approximately 10 days. After 10 days, the colonies were fixed using formaldehyde and then stained with 0.1% crystal violet (Vicmed, China). These colonies were subsequently photographed and counted visually.

### EdU proliferation assay

The EdU proliferation assay was performed using an EdU kit (Beyotime, Shanghai, China). Briefly, cells were inoculated in 96-well plates at a density of 1 × 10^4^ cells per well, then cultured for 12 h and incubated with EdU for 2 h. The rest steps are performed according to the manufacture’s instructions. After staining for nucleic acids with DAPI (Beyotime, Shanghai, China), images were acquired by using an inverted fluorescence microscope (Nikon, Tokyo, Japan).

### Transwell assay

The invasion and migration assay were performed using trans-well chambers (Corning, USA) pre-covered or uncovered with Matrigel (BD Biosciences, USA), respectively. Briefly, 5 × 10^4^ cells were seeded onto the upper chamber, while 600ul medium with 10% FBS was provided in the lower chamber. After 24-36 h of incubation, cells on the upper membrane surface were scraped off, the invaded and migrated cells were fixed with methanol and stained with 0.1% crystal violet for 30 min. Images were acquired by using an inverted microscope.

### Wound healing assay

Cells were seeded onto 6-well plates. When the confluence of cells reached more than 80%, the monolayers were scratched by a 200 μL pipette, and the cell debris was removed by washing with PBS. After that, cells were cultured with medium without FBS. Images of cell migration were captured at the same locations at 0 h, 24 h and 48 h.

### Cell cycle and apoptosis analysis

Cell cycle and apoptosis analysis were performed as we described before [15].

### RNA pull-down assay and mass spectrometry

RNA pull-down was performed using a kit (Thermo Fisher Scientific, CA, USA). The biotinylated circPAK1 probe and control probe were designed by RiboBio (Guangzhou, China), and the probe sequences were listed in Table S5. Approximately 1 × 10^7^ LM3 cells were lysed and then incubated with biotinylated probes for 4 h at 4 °C. After that, incubated with 50 μL of streptavidin-coated magnetic beads at room temperature for 1 h. Then, the beads-probe-protein complex was washed with wash buffer. The retrieved proteins were boiled in a SDS buffer and separated using SDS-PAGE, followed by staining with fast silver stain kit (Beyotime, Shanghai, China). The protein bands in circPAK1 probe group in comparison with control group were analyzed by Q Exactive mass spectrometer (Thermo Fisher Scientific, CA, USA).

### RNA immunoprecipitation (RIP) assay

RIP was performed using Magna RIP RNA Binding Protein Immunoprecipitation Kit (Millipore, MA, USA) according to the manufacturer’s protocols. Antibodies including anti-IgG (Millipore, MA, USA), anti-YAP (Proteintech, Wuhan, China) and anti-14-3-3ζ(Proteintech, Wuhan, China) were listed in Table S4. The relative interaction between circRNA and proteins were assessed by qPCR, and normalized to input.

### Co-immunoprecipitation (co-IP) assay

To evaluate the binding between LATS2, YAP and 14–3-3ζ, proteins were immunoprecipitated by using a IP kit (Thermo Fisher Scientific, CA, USA) according to the manufacture’s protocols and then assessed by immunoblotting.

### Cytoplasm and nucleus fractionation

Cytoplasm and nucleus fractionation was performed with Nuclear and Cytoplasmic Protein Extraction Kit (Beyotime, Shanghai, China) in accordance with the manufacturer’s instructions (Beyotime, Shanghai, China). Cytoplasm and nucleus fractionations of protein and RNA were analyzed by western blot and qPCR respectively, to determine the level of YAP.

### Fluorescence in-situ hybridization (FISH)

Briefly, after the fixation and permeabilization of HCC cells, Hybridization with circPAK1 probe (Sequence is shown in Table S5) (RiboBio, Guangzhou, China) was performed with a Fluorescent in Situ Hybridization Kit (RiboBio, Guangzhou, China) in accordance with the manufacturer’s instructions. Fluorescence images were acquired using a confocal laser scanning microscopy (Zeiss, Jena, Germany).

### RNA sequencing

To identify differentially expressed circRNAs, circRNA sequencing was performed using 3 paired HCC tissues and adjacent normal tissues. The tissue quality control, sample preparation and circRNA sequencing were performed by Huada Gene (Shenzhen, China). To explore the influence of circPAK1 knockdown on global gene expression profiles, circPAK1-knockdown and control LM3 cells were lysed in Trizol. The RNA sequencing was analyzed by Tiangen Biotech (Beijing, China).

### Exosome isolation

Exosomes were isolated from CM by differential ultracentrifugation. Briefly, cells were cultured in DMEM with 10% exosome-free FBS (Absin, Shanghai, China). When the confluence of cells reached more than 80%, CM was collected and centrifuged at 300 g for 10 min. Subsequently, cells and debris were removed through 2 × 10^3^ g centrifugation for 20 min and 1 × 10^4^ g centrifugation for 30 min. The resulting supernatant was pre-cleared through 0.22 μm filters (Millipore, MA, USA), followed by ultra-centrifugation at 1× 10^5^ g for 70 min. Repeat the previous step and the exosomes can be collected. For exosomal RNA extraction, exosomes were pre-treated with RNase, and an equal number of exosomes were used for RNA extraction.

### Transmission electron microscopy (TEM)

Exosomes were examined by electron microscopy using negative staining and quantified using the NanoSight NS300 instrument (Malvern Instruments Ltd. UK) equipped with NTA 3.0 analytical software (Malvern Instruments Ltd. UK).

## Results

### CircPAK1 is highly expressed in HCC

**Fig. 1:**
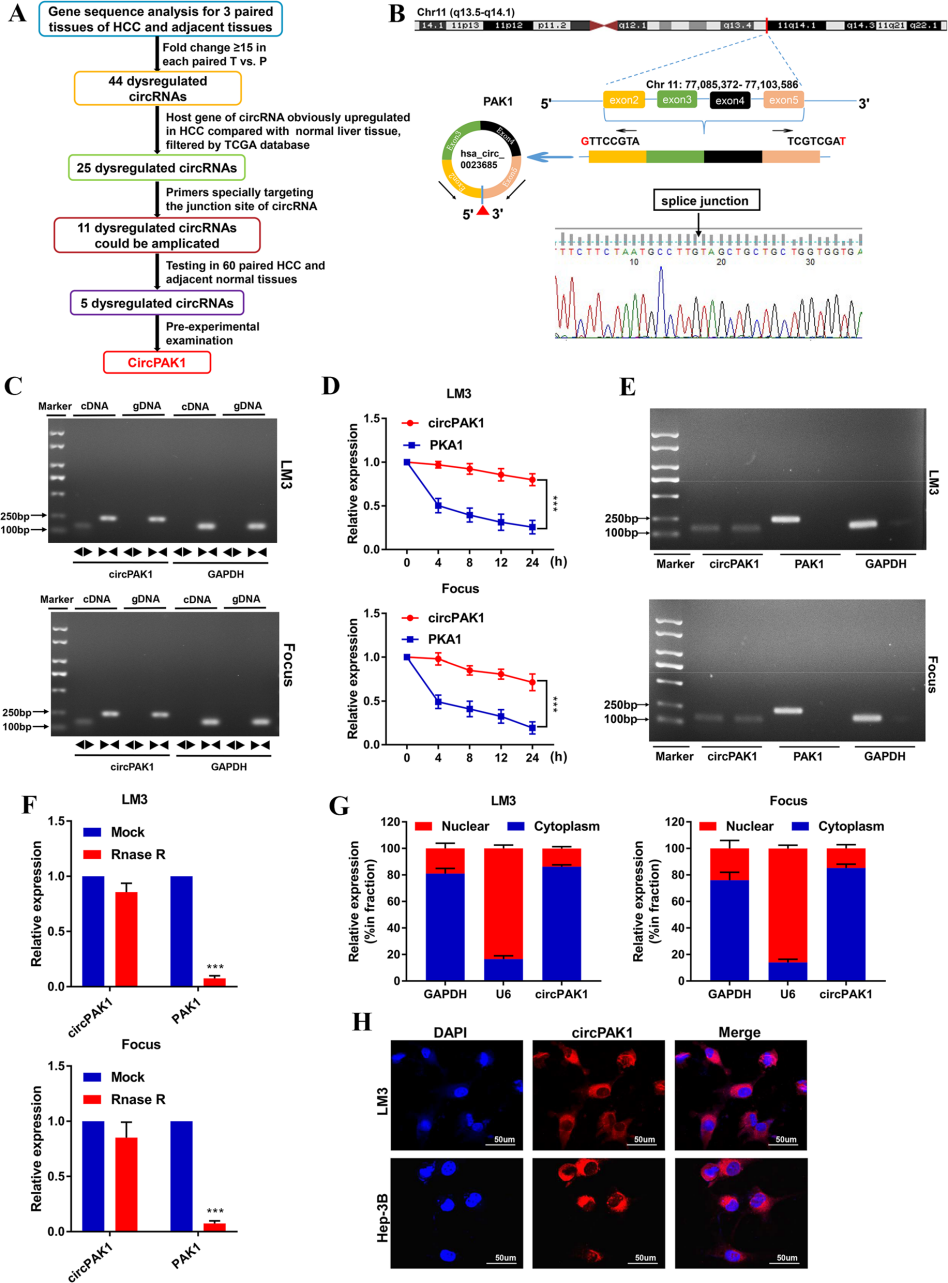
Identification of circPAK1. **A** Flow diagram of circRNAs screening in 3 pairs HCC tissue. **B** Illustration of the annotated genomic region of circPAK1 Sanger sequencing was conducted to confirm the ‘head-to-tail’ splicing of circPAK1. **C** The divergent primers detected circPAK1 in cDNA but not in gDNA, GAPDH was used as a negative control. **D** Relative RNA level of circPAK1 and linear PAK1 in different time point. **E** and **F** showed the relative RNA level of circPAK1, linear PAK1 and GAPDH treated with RNase R. **G** The nuclear mass separation assay and **H** FISH showed that the sub-cellular distribution of circPAK1 was mostly present in the cytoplasm. *p < 0.05; **p < 0.01; ***p < 0.001. Data were shown as mean ± SEM

To identify circRNAs involved in HCC progression, high-throughput sequencing was performed on 3 paired tissues of HCC and adjacent tissues. Among the 351 differential expressed circRNAs (fold change≥2 and q value≤0.001)，a total of 211 were upregulated and 140 were downregulated in the HCC tissues than their paired normal tissues. As shown in Fig. 1A, from the circular RNA sequencing results, 44 differentially expressed genes (DEGs) with fold change ≥15 were identified, all higher expressed in tumor tissues than those in normal tissues. The 44 upregulated circRNAs were listed in Table S6. Usually, the levels of circRNAs are in accordance with their respective mRNA levels [16]. Therefore, 25 DEGs whose host genes are significantly upregulated in HCC tissues were selected from these 44 DEGs by using The Cancer Genome Atlas (TCGA) database. We designed specific primers targeting the junction site of these 25 DEGs, respectively, and only 11 circRNAs could be successfully amplified by qRT-PCR. Then, we examined the expression of these 11 circRNAs by qRT-PCR in 20 HCC tissues and matched adjacent normal tissues, and only 5 DEGs were statistically significant. As the *P* value of paired comparison of circPAK1 is the most significant, so circPAK1 was eventually selected for the subsequent study (Fig. S1A).

CircPAK1 is derived from chromosome 11: 77085372–77,103,586 and consists of 4 adjacent exons in the PAK1 gene, sanger sequencing confirmed the junction site of circPAK1 (Fig. 1B). To further identify the ring structure, convergent and divergent primers were designed, and the PCR product was determined by agarose gel electrophoresis (Fig. 1C). Additionally, the RNase R digestion and actinomycin D RNA stability assays confirmed the stability of circPAK1 compared with PAK1 (Fig. 1E, F). The FISH and qRT-PCR results suggested that the localization of circPAK1 was mostly in cytoplasm (Fig. 1G, H).

**Fig. 2:**
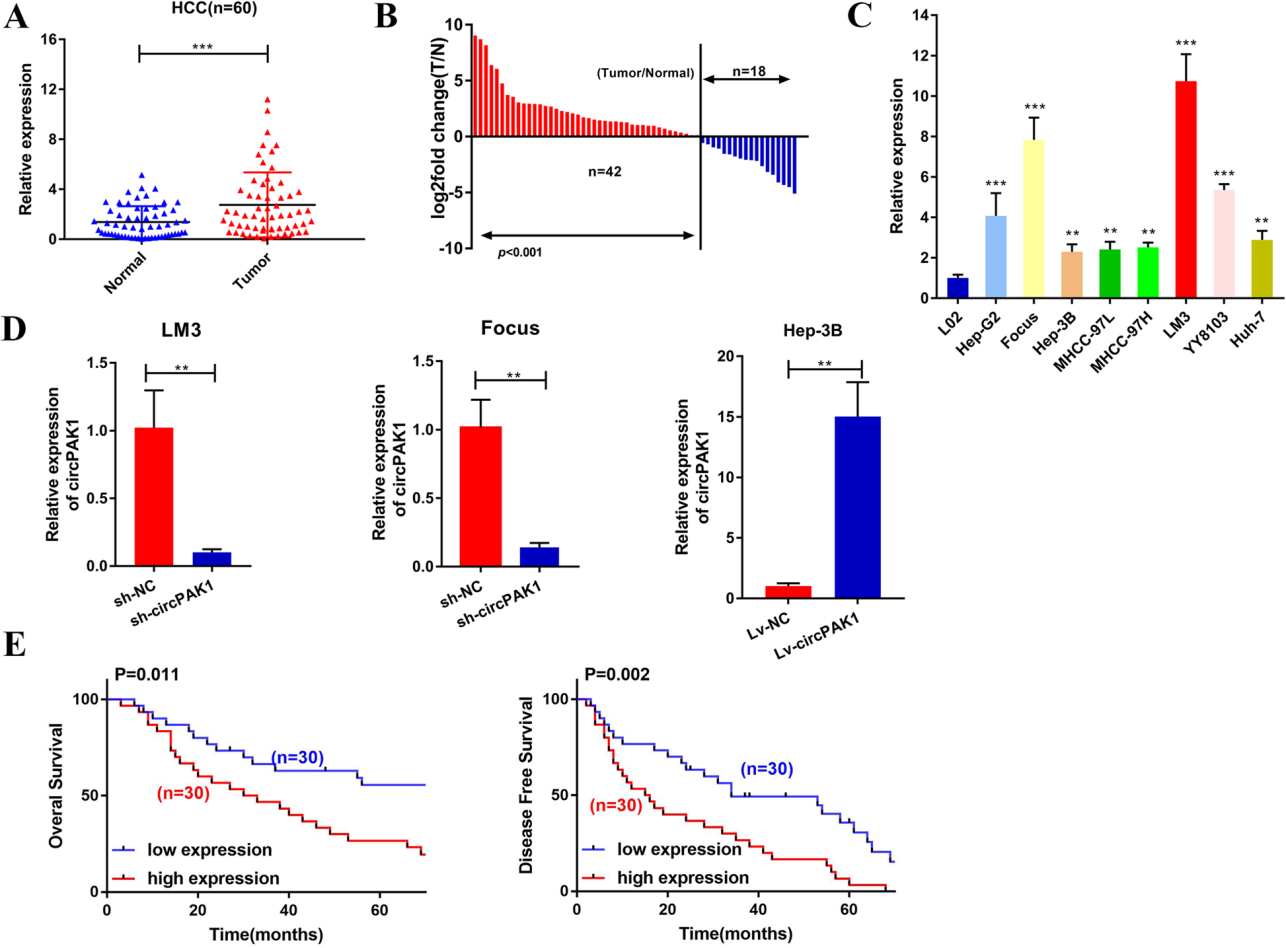
Characteristics of circPAK1 in HCC. **A** Differential expression of circPAK1 in human HCC tissues and adjacent nontumor tissues of 60 patients. **B** Relative expression of circPAK1 in 60 pairs of HCC tissues and matched normal tissues, shown as log2 (Tumor/Normal). **C** Relative expression of circPAK1 in HCC cell lines was assessed by qRT-PCR. **D** LM3 and Focus cells were transfected with circPAK1 shRNA (sh-circPAK1) or control shRNA (sh-NC); Hep-3B cells were infected with circPAK1 overexpression lentivirus (Lv-circPAK1) or control lentivirus (Lv-NC), the transfection efficiency was assessed by qRT-PCR. **E** Elevated expression of circPAK1 was negatively correlated with the overall survival (OS) and disease-free survival (DFS) of HCC patients. *p < 0.05; **p < 0.01; ***p < 0.001. Data were shown as mean ± SEM

The levels of circPAK1 in 60 pairs of HCC tissues and adjacent non-tumor tissues were determined by qRT-PCR. The results showed that circPAK1 was significantly overexpressed in HCC tissues than normal liver tissues (Fig. 2A, B). Besides, circPAK1 was also overexpressed in HCC cell lines compared with normal hepatocytes (L02) (Fig. 2C). For the subsequent study, we chose LM3 and Focus cells to establish circPAK1 stable knockdown cell lines by infection with shRNA specially targeting the junction sites of circPAK1, while Hep-3B to infect with a lentiviral vector to establish circPAK1 stable overexpression cell lines. As shown in Fig. 2D, stable circPAK1-knockdown and circPAK1-overexpression cell lines were successfully established. To evaluate the clinical value of circPAK1, the 60 HCC patients were divided into two groups based on the median expression of circPAK1 (Table 1). Our data suggested that higher expression of circPAK1 was more likely to develop larger tumor size, higher risks of LN metastasis, advanced TNM stage and microvascular invasion (MVI) (all *P* values<0.05); however, no statistical significance for age, gender, AFP, PIVKA-II, HBsAg status, tumor multiplicity, differentiation and vascular invasion was observed. Importantly, patients in the high expression group showed poor overall survival and disease-free survival than the patients in the low expression group (Fig. 2E). Taken together, these results demonstrate that circPAK1 is abundant and upregulated in HCC tissues and cell lines, and it could serve as a valuable molecular biomarker for HCC.

**Table 1:** The clinicopathological relevance analysis of circPAK1 expression in HCC patients

		Low group	High group	*P* value
Characteristics		(*N* = 30)	(*N* = 30)	
Age (years)	< 50	10	12	0.592
	≥50	20	18	
Gender	Male	16	18	0.602
	Female	14	12	
HBsAg status	Negative	22	23	0.766
	Positive	8	7	
AFP (ng/ml)	< 400	12	7	0.165
	≥400	18	23	
PIVKA-II	< 50	13	9	0.284
	≥50	17	21	
Tumor multiplicity	Single	19	16	0.432
	Multiple	11	14	
Tumor diameter (cm)	≤5	17	8	0.018*
	>5	13	22	
Differentiation	Well to moderate	19	15	0.297
	Poor	11	15	
Lymph node metastasis	Negative	19	10	0.019*
	Positive	11	20	
TNM stage	I + II	18	10	0.038*
	III + IV	12	20	
MVI	Negative	17	9	0.037*
	Positive	13	21	

Annotation: *MVI* Microvascular invasion. *P < 0.05

### CircPAK1 promotes HCC proliferation, migration and invasion

**Fig. 3:**
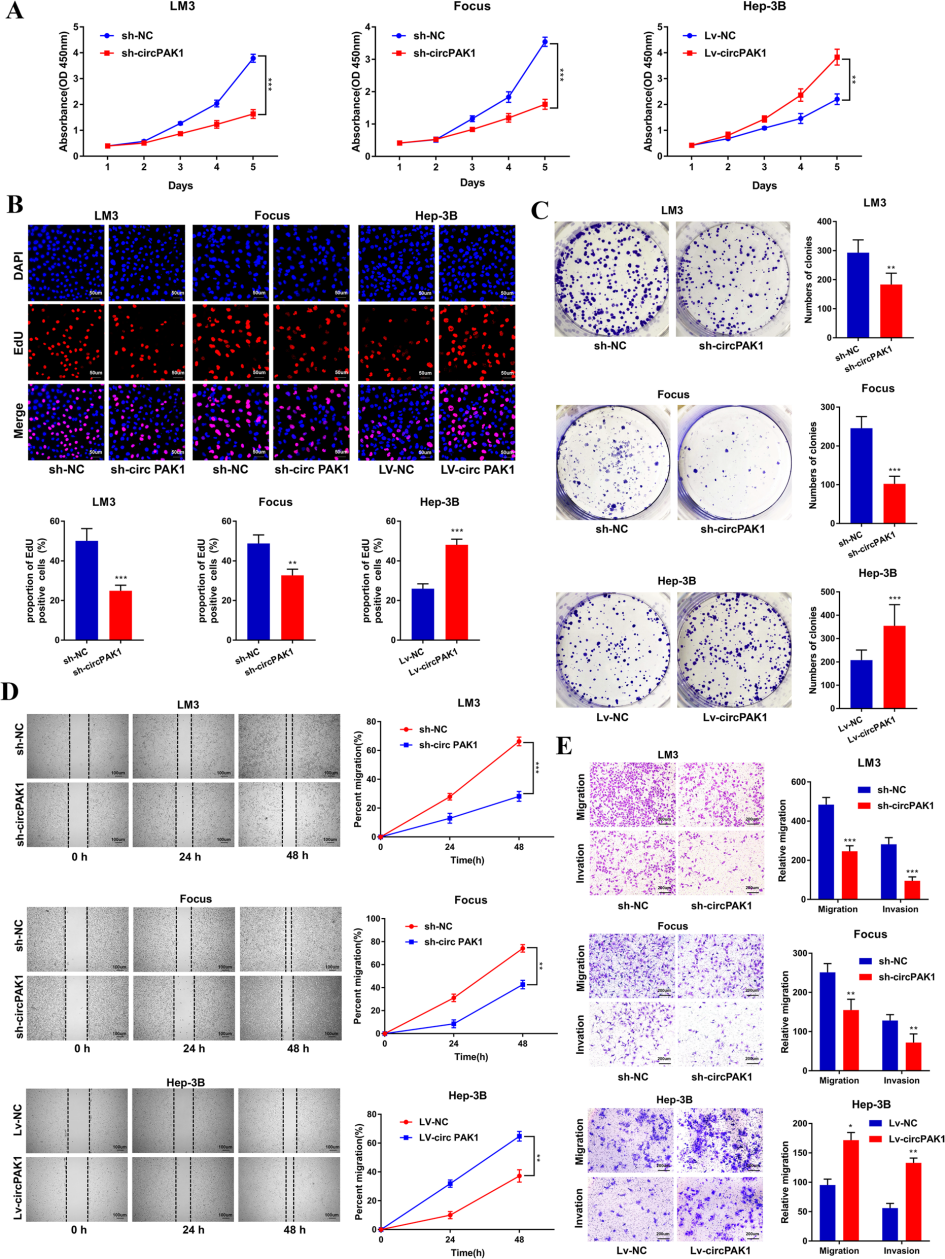
CircPAK1 promotes HCC proliferation, migration and invasion. **A** The proliferation of sh-circPAK1 or Lv-circPAK1 cells was determined by CCK8 assay. **B** EdU incorporation of sh-circPAK1 or Lv-circPAK1 cells was decided, scale bar, 50 μm. **C** The colony formation of sh-circPAK1 or Lv-circPAK1 cells was determined. **D** Cell migration ability was evaluated by wound healing assay, scale bar, 100 μm. **E** Invasive or migrated cells were measured by transwell assay with or without matrix, scale bar, 200 μm. *p < 0.05; **p < 0.01; ***p < 0.001. Data were shown as mean ± SEM

To explore the effect of circPAK1 on cell proliferation, CCK-8, EdU and colony formation assays were performed, and the results showed that circPAK1 knockdown significantly attenuated the growth of LM3 and Focus cells, while converse results were observed in circPAK1 overexpression cell lines (Fig. 3A, B and C). To further investigate the effect of circPAK1 on HCC cell migration and invasion, wound-healing and transwell assays were performed. The results revealed that circPAK1 inhibition could significantly reduce the migration and invasion ability of LM3 and Focus cells, while circPAK1 overexpression markedly increased these abilities (Fig. 3D, E). Taken together, these results indicated that circPAK1 enhances HCC cell proliferation and motility.

### CircPAK1 inhibits HCC apoptosis

**Fig. 4:**
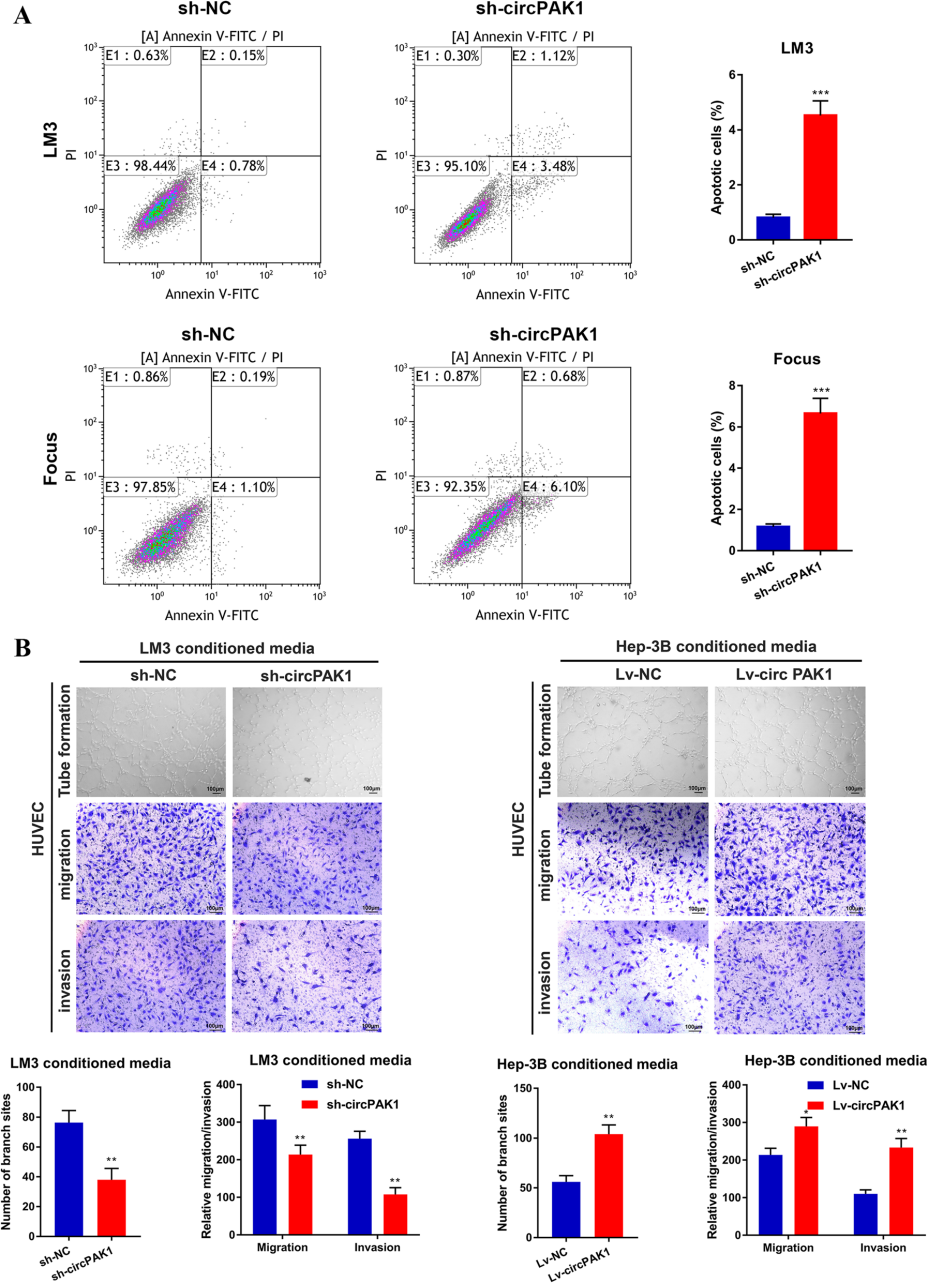
CircPAK1 promotes the apoptosis and angiogenesis of HCC cells. **A** The percentage of apoptotic cells of STK39 stable knockdown or overexpression cells was analyzed by Annexin V-FITC/PI staining assay. **B** HUVEC tube formation, migration and invasion were evaluated using the conditioned medium (CM) from LM3 (circPAK1 knockdown) and Hep-3B (circPAK1 overexpression) cells, scale bar, 100 μm. *p < 0.05; **p < 0.01; ***p < 0.001. Data were shown as mean ± SEM

We also explored whether circPAK1 could regulate the cell cycle and apoptosis. We found that the intervention of circPAK1 did not affect the cell cycle (Fig. S1B). However, it is worth noting that circPAK1 knockdown promoted cell apoptosis in both LM3 and Focus cells (Fig. 4A). These results suggest that the promotion ability of circPAK1 on HCC cell growth may depend on inhibiting tumor apoptosis.

### CircPAK1 promotes angiogenesis of HCC in vitro

Tumor cells can promote angiogenesis by modifying the tumor microenvironment, contributing to early progression, invasion, postoperative recurrence, and metastasis of HCC [17, 18]. Therefore, the ability to promote angiogenesis is also one of the important indicators to measure tumor invasiveness. Then, we performed an angiogenesis experiment to study whether circPAK1 has a promoting effect on tumor angiogenesis.

HUVEC tube formation, migration and invasion were evaluated using the conditioned medium (CM) from LM3 (circPAK1 knockdown) and Hep-3B (circPAK1 overexpression) cells. As shown in Fig. 4B, CM from circPAK1 knockdown cells significantly inhibit the migration and invasion of HUVEC cells. In contrast, the opposite results were found in circPAK1 overexpression cells. As envisioned, the tube formation assay also showed that the number of branch sites was decreased in HUVECs treated with CM from circPAK1 knockdown cells compared with that from control cells. The above abilities of HUVECs were enhanced after incubation with CM from circPAK1 overexpression cells. Taken together, these findings suggest that circPAK1 could induce angiogenesis of HCC in vitro.

### CircPAK1 enhances in vivo HCC tumor growth and metastasis and CS/si-circPAK1 nanocomplexes could effectively inhibit these abilities

**Fig. 5:**
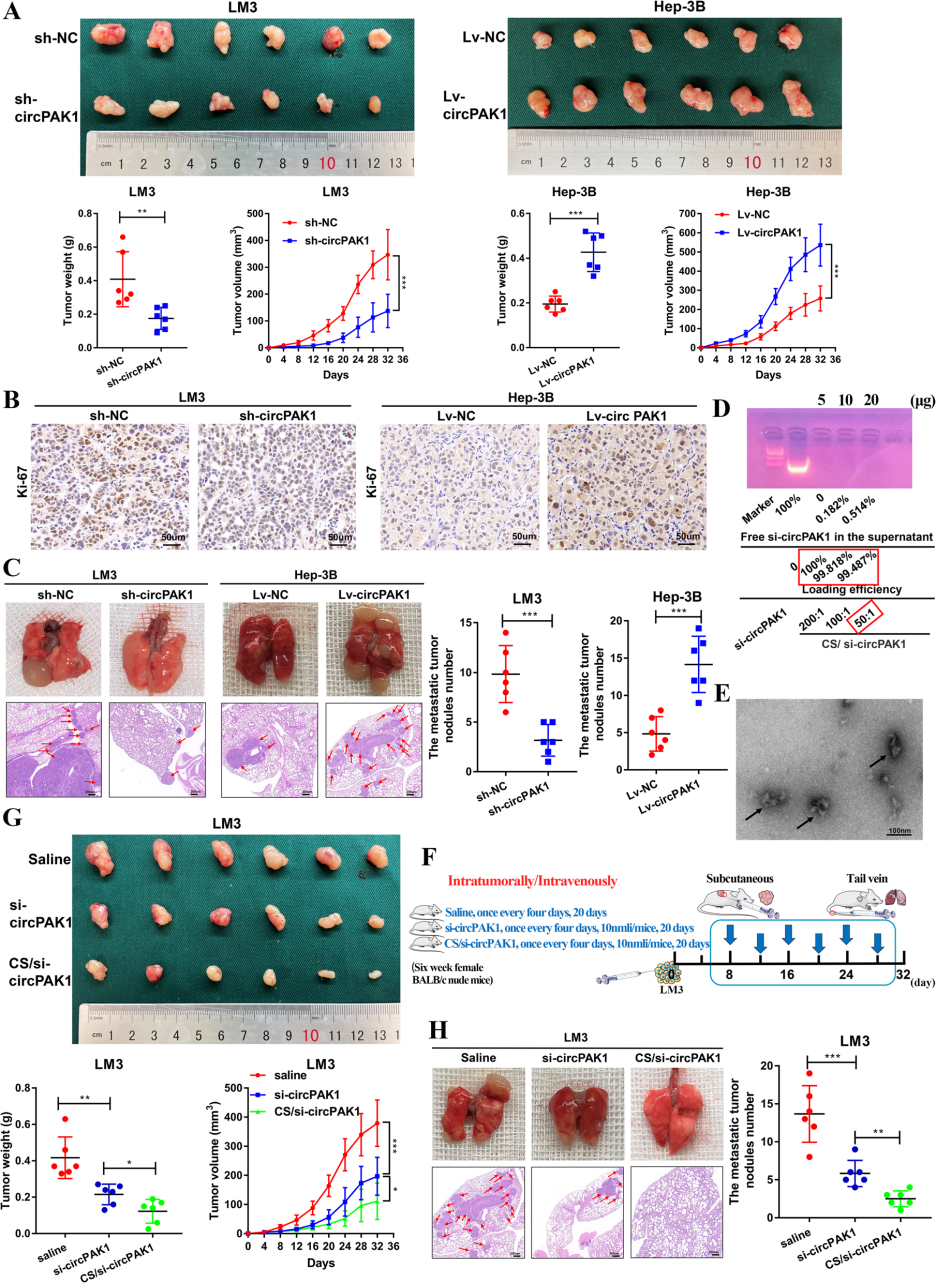
CircPAK1 enhances HCC tumor growth and metastasis in vivo and CS/si-circPAK1 nanocomplexes inhibits HCC growth and metastasis in vivo. **A** Nude mice were subcutaneously injected with circPAK1 stable knockdown or overexpression cells. The tumor volume and average weight were determined. **B** IHC analysis of Ki-67 in the tumors derived from mice, scale bar, 50 μm. **C** CircPAK1 stable knockdown or overexpression cells were injected into the tail vein of nude mice to induce lung metastasis, and gross lung tissues were obtained; liver metastatic nodules were subjected to HE staining as indicated, scale bar, 200 μm. **D** The loading efficiency of CS. **E** TEM images of CS/si-circPAK1 nanocomplexes (CS/ si-circPAK1 = 50/1). **F** The flow diagram showed the scheme of intratumorally/intravenously with saline, si-circPAK1 or CS/si-circPAK1 into mice. **G** and **H** Weight volume and weight change after 20 days of treatment with saline, si-circPAK1 or CS/si-circPAK1 for xenograft tumors or lung metastasis models. The tumor volume and average weight were determined; gross lung tissues were obtained and liver metastatic nodules were subjected to HE staining as indicated, scale bar, 200 μm. *p < 0.05; **p < 0.01; ***p < 0.001. Data were shown as mean ± SEM

To assess the in vivo effect of circPAK1 on HCC growth and metastasis, we then generated tumor xenograft and lung metastasis models. The xenograft tumor model showed that no matter the tumor size or the tumor weight was significantly decreased in the sh-circPAK1 group, while increasing in the Lv-circPAK1 group (Fig. 5A). The results of IHC staining also showed that the level of Ki-67 was lower in the sh-circPAK1 group and higher in the Lv-circPAK1 group (Fig. 5B). Additionally, the lung metastasis model showed a similar trend as the results of xenograft model, for there were less metastasis foci in the sh-circPAK1 group, and more in the Lv-circPAK1 group (Fig. 5C). These results indicate that circPAK1 could promote tumor growth and metastasis of HCC.

Based on the new understanding of tumor pathogenesis, gene targeting therapy in cancer has attracted a wide range of attention and proved to be a rational approach in cancer therapy. Chitosan (CS)-based materials have a long history as drug delivery vehicles due to their characteristics of non-toxicity, biodegradability, high drug carrying capability, high plasma membrane permeability, including transport across mucosal membranes, the pH-dependent release of therapeutic agents, multi-functionality, and suitable circulation time [19–21]. We established three nanocomplexes in different proportions based on the weight ratios of CS and si-circPAK1. The loading efficiency of chitosan to si-circPAK1 was more than 99% under these three ratios (Fig. 5D). Therefore, to ensure the loading efficiency, we choose the largest chitosan utilization ratio-CS/si-circPAK1 of 50. The TEM (Fig. 5E) and particle size potentiometer (Fig. S2A) revealed that the particle size of CS/si-circPAK1 nanocomplexes was on an average of 101.9 nm. The zeta potential was − 21.4 and + 31.6 mV (Fig. S2B). By simulating the temperature and pH of human body to detect the release of siRNA from CS, we found that siRNA can be released persistently and efficiently (Fig. S2C).

To study the effect of CS/si-circPAK1 nanocomplexes on the proliferation and metastasis of HCC in vivo, we generated several groups of subcutaneous xenograft tumor and lung metastasis models. Figure 5F shows the injection scheme with saline, si-circPAK1 or CS/si-circPAK1 nanocomplexes into mice. We noticed that no matter the tumor size or the weight in the CS/si-circPAK1 nanocomplexes treated group was much smaller and lighter than the saline-treated group, even better than the si-circPAK1 group (Fig. 5G). IHC staining of tumors from CS/si-circPAK1 nanocomplexes treated group also revealed a significant decrease of Ki67 (Fig. S2D). Furthermore, the lung metastasis models from CS/si-circPAK1 nanocomplexes treated group showed a better inhibition effect on lung metastasis lesions (Fig. 5H). Collectively, these findings suggest that circPAK1 could promote the in vivo growth and metastasis of HCC, and the CS/si-circPAK1 nanocomplexes could effectively inhibit these abilities.

### CircPAK1 facilitates the progression of HCC through hippo signaling pathway by promoting nucleus transport of YAP

**Fig. 6:**
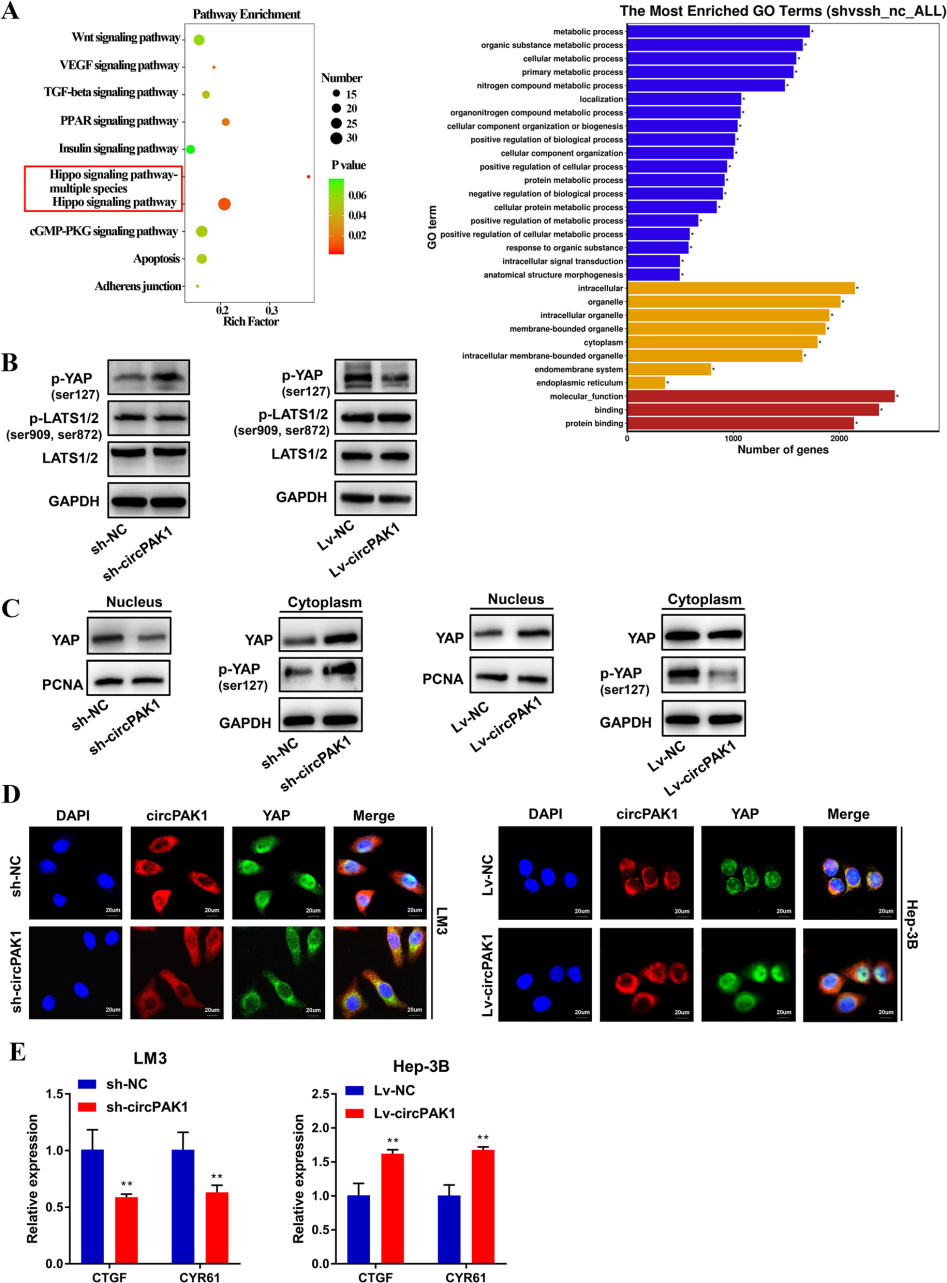
CircPAK1 mediates oncogenic effects on HCC through inactivating Hippo signaling pathway. **A** Pathway enrichment analysis of differentially expressed genes in RNA-sequence data. **B** Levels of p-YAP (ser127), LATS1/2 and p-LATS1/2 were examined by western blotting in circPAK1 stable knockdown or overexpression cells. **C** The expression of YAP1 protein in nuclear and cytoplasmic component of circPAK1 stable knockdown or overexpression cells. **D** IF assay in stable circPAK1-knockdown LM3 cells or stable circPAK1-overexpressed Hep-3B cells, scale bar, 20 μm. **E** The level of downstream target gene of YAP, CTGF and CYR61, were determined by qRT-PCR. *p < 0.05; **p < 0.01; ***p < 0.001. Data were shown as mean ± SEM

To explore the molecular mechanisms of circPAK1 involved in HCC development, we performed RNA-sequencing. By listing the top 10 of pathway enrichment, a strong correlation between the Hippo signaling pathway and the downstream of circPAK1 is shown (Fig. 6A). The hippo signaling pathway is highly conserved in evolution, and it can regulate organ size by regulating cell proliferation and apoptosis [22, 23]. The dysregulation of Hippo signaling pathway will cause uncontrolled proliferation of cells, thus leading to the overgrowth of tissues and organs [24]. Several studies have pointed out that the inactivation of Hippo signaling pathway was closely correlated with the progression of HCC [25–27].

Combined with the above tumor-promoting phenotype of circPAK1 and the result of RNA-seq, we, therefore, explored the effect of circPAK1 on Hippo signaling pathway. We first performed a western blot to analyze the changes in proteins related to Hippo signaling pathway. We found that neither the knockdown nor the overexpression of circPAK1 did not affect the level of LATS1/2 or its phosphorylation level (Fig. 6B); however, p-YAP was increased in the circPAK1 knockdown group, while decreased in the circPAK1 overexpression group. Cytoplasmic retention of YAP plays a vital role in the Hippo pathway-mediated control of cell proliferation and apoptosis. It is well established that non-phosphorylated YAP can be transported into the nucleus and interact with TEAD1–4 to activate multiple downstream target genes, which is very important to enhance tumor proliferation and apoptosis inhibition [28–30]. However, YAP (p-YAP) phosphorylation by p-LATS1/2 will be sequestrated in the cytoplasm and eventually degraded [31].

Based on the above perceptions, we speculate that circPAK1 may promote the nucleus transport of YAP and then affect the proliferation, invasion and metastasis of HCC. Subsequently, we conducted the cytoplasm and nucleus fractionation assay to observe the effect of circPAK1 on the localization of YAP. As shown in Fig. 6C, overexpression of circPAK1 promoted the nucleus localization of YAP, while reducing the p-YAP (ser-127) level. The opposite results were observed under the condition of circPAK1 knockdown. Meanwhile, immunofluorescence also confirmed this observation (Fig. 6D). These findings suggest that circPAK1 could guide the nucleus transportation of YAP. Besides, the main downstream target gene of YAP, CTGF and CYR61, were downregulated when circPAK1 knockdown, but upregulated when circPAK1 overexpression (Fig. 6E).

We further explored whether the tumor-promoting effect of circPAK1 is dependent on YAP by treating the circPAK1 overexpression Hep-3B cells with YAP siRNA. Fig. S3A shows the efficiency of YAP knockdown. As envisioned, the tumor promoting effect of circPAK1 on HCC was significantly weakened (Fig. S3B-F). Taken together, our data revealed that circPAK1 enhances HCC progression by accelerating YAP nucleus transport, which leads to the inactivation of Hippo signaling pathway.

### CircPAK1 competitively binds 14–3-3ζ with YAP thus promoting the nucleus transportation of YAP

As a transcription coactivator, the nucleus transportation of YAP will directly determine whether it can interact with TEAD1–4 to activate multiple downstream target genes [28–30]. There have been many studies on downstream signals of YAP after its nucleus implantation, but little is known about by what mechanism YAP is regulated before its transportation to the nucleus.

Serving as miRNA sponge is one of the most common biological functions of circRNA, while binding with argonaute 2 (AGO2) RNA is the vital basis for circRNA to act as competitive endogenous RNA (ceRNA). However, RIP-qPCR assay showed that circPAK1 couldn’t be enriched in AGO2 antibody complexes compared with anti-IgG, so acting as ceRNA of circPAK1 was excluded (Fig. S4A).

**Fig. 7:**
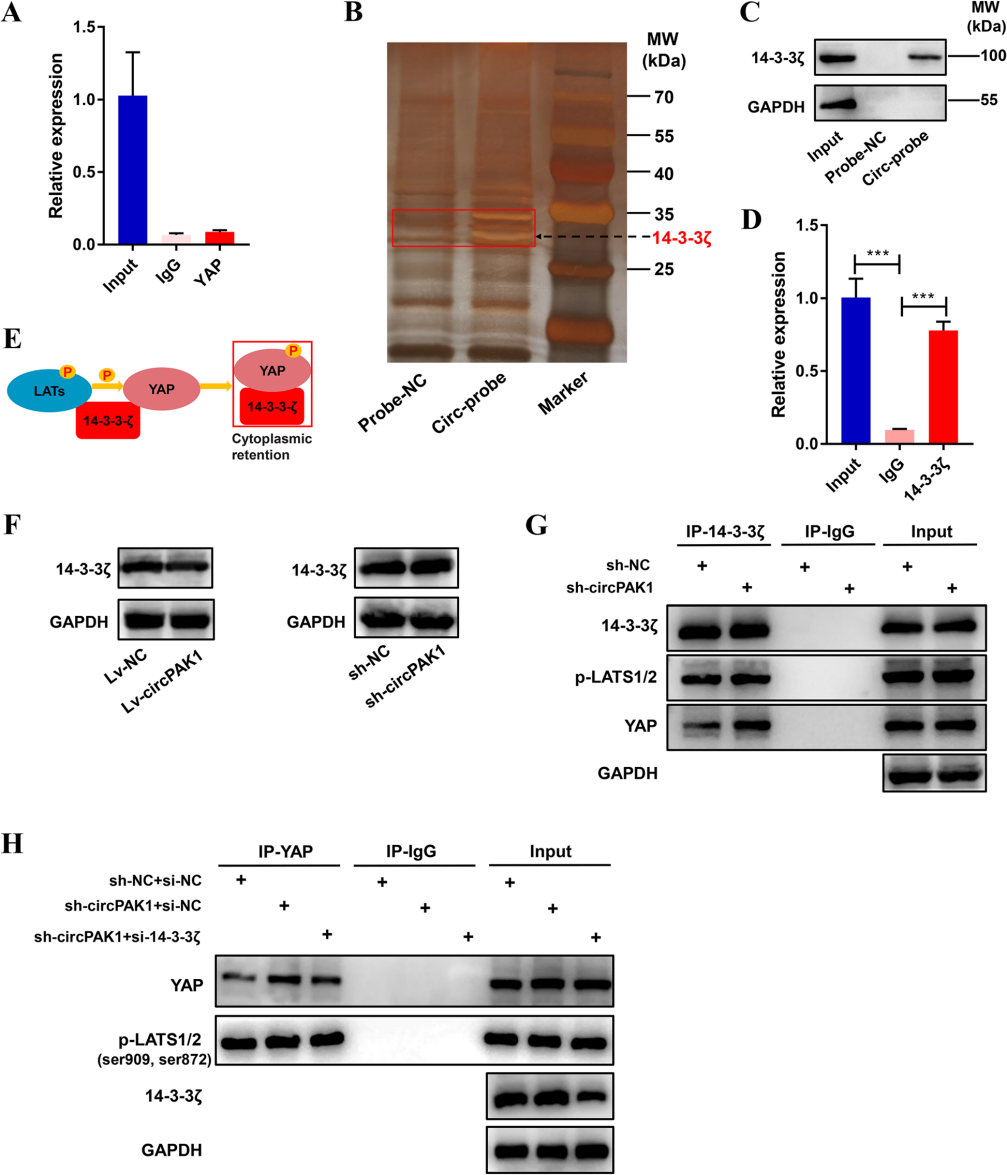
CircPAK1 competitively binds 14–3-3ζ with YAP thus reduces the phosphorylation of YAP by p-LATs. **A** RIP-qPCR was performed to explore the association between circPAK1 and YAP. **B** Silver-stained SDS-PAGE gel-containing proteins derived from RNA pulldown by circPAK1 probe and negative control. The red rectangle was used for mass spectrometric analysis. **C** Western blot analysis showed the specific association of circPAK1 with 14–3-3ζ. **D** qRT-PCR analysis of the RNAs derived from the RIP assays. **E** A proposed model for 14–3-3ζ mediation of p-LATS-YAP interactions. **F** Western blot analysis identified 14–3-3ζ protein expression level in LM3 transfected sh-circPAK1. **G** CircPAK1 stable knockdown LM3 cells were subjected to IP using 14–3-3ζ antibody or control IgG, followed by IB with 14–3-3ζ, p-LATS1/2 and YAP antibody, to confirm and determine the 14–3-3ζ- p-LATS-YAP protein complex. **H** CircPAK1 stable knockdown LM3 cells were subjected to IP using YAP antibody or control IgG, followed by IB with 14–3-3ζ, p-LATS1/2 and YAP antibody. *p < 0.05; **p < 0.01; ***p < 0.001. Data were shown as mean ± SEM

It has been reported that circRNA can facilitate YAP nucleus transport by binding YAP protein directly, thus promoting the metastasis of colorectal cancer [32]. To verify whether circPAK1 can directly bind to YAP, we also performed RIP-qPCR and found that the result was not consistent with our hypothesis (Fig. 7A). So, how does circPAK1 affect the cellular spatial localization of YAP? We speculate that another mediator protein mediates the association between circPAK1 and YAP. Next, we performed an RNA-pulldown assay and mass spectrometry analysis to identify potential circPAK1-interacting proteins in LM3 cells. Surprisingly, 14–3-3ζ protein was identified as the main protein using silver staining (Fig. 7B) and liquid chromatography mass spectrometry (Fig. S4B). WB (Fig. 7C) and RIP were also performed and confirmed this specific binding (Fig. 7D).

Nucleus, import/export of YAP, is strictly regulated by 14–3-3ζ, as evidenced by the fact that 14–3-3ζ assembles with YAP, isolates it in the cytoplasm and prevents it from further signal amplification [33]. Therefore, the direct or indirect change of 14–3-3ζ level will affect the spatial localization of YAP in a cell. It has been proved that 14–3-3ζ can recruit p-LATS and YAP to form a complex, which can induce the phosphorylation of YAP and lead to its cytoplasmic retention, and 14–3-3ζ is the key regulatory factor of this complex (Fig. 7E) [34]. In addition, 14–3-3ζ can be dissociated from non-phosphorylated YAP under the condition of hypoxia, thus leading to the nucleus transport of YAP [35]. These studies showed that the cytoplasmic fixation of 14–3-3ζ on YAP is based on the non-phosphorylation of YAP. We hypothesized that circPAK1 could directly or indirectly affect the level of 14–3-3ζ by interacting with 14–3-3ζ, thus affecting the recruitment of P-LATs and YAP, then further inhibiting the phosphorylation of YAP, and finally, exerting the tumor promoting effect by facilitating the nucleus transport of YAP. Interestingly, we found that circPAK1 did not affect the 14–3-3ζlevel, suggesting that the level of 14–3-3ζmay be affected indirectly (Fig. 7F). To approach this, we first performed immunoprecipitation assay in circPAK1-knockdown LM3 cells. The existence of 14–3-3ζ- p-LATS-YAP protein complex was confirmed, and the interaction between p-LATS and YAP in the circPAK1-knockdown group was significantly increased compared with the sh-NC group, while significantly decreased in the circPAK1-overexpression group (Fig. 7G, Fig. S4D). Next, we silenced 14–3-3ζ in circPAK1 stable knockdown cell lines to explore whether the interaction between p-LATS and YAP can still be influenced by the change of circPAK1 level. The silencing efficiency of si-14-3-3ζ was shown in Fig. S4C. Surprisingly, we found that the increased interaction between p-LATS and YAP was significantly reversed after 14–3-3ζ knockdown (Fig. 7H). The immunofluorescence also showed that the nucleus location of YAP was restored after the knockdown of 14–3-3ζ (Fig. S4E). Taken together, the promotion of YAP nucleus transport mediated by circPAK1 is through binding with 14–3-3ζ, which weakens the recruitment of p-LATs and YAP, and then reduces the phosphorylation of YAP by p-LATs.

### CircPAK1 is upregulated in lenvatinib-resistant HCC cells and exosome-mediated circPAK1 transfer and transmission of lenvatinib resistance

**Fig. 8:**
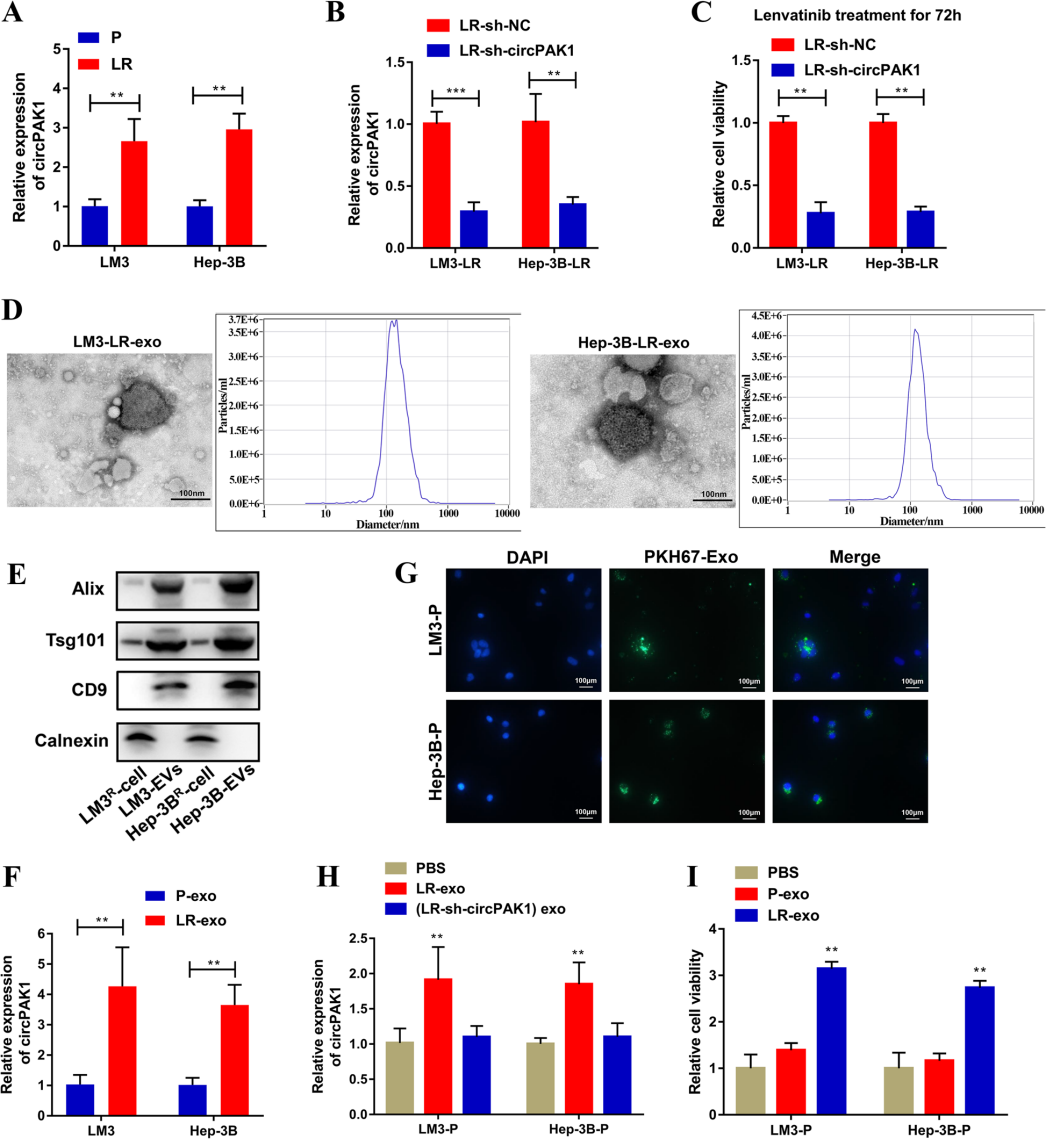
CircPAK1 transmits lenvatinib resistance by exosomes. **A** The level of circPAK1 in the two lenvatinib-resistant HCC cell lines was assessed by qRT-PCR. **B** The knockdown efficiency of circPAK1 in the two lenvatinib-resistant HCC cell lines was assessed by qRT-PCR. **C** The effect of circPAK1 knockdown on lenvatinib resistance was assessed by CCK8 assay. **D** TEM and NTA of exosomes isolated from LM3-LR and Hep-3B-LR culture media, scale bar, 100 nm. **E** Exosomal protein positive markers (Alix, Tsg101 and CD9) and negative marker (Calnexin) detection by western blot from purified exosomes and exosome-depleted cell extracts. **F** qRT-PCR analysis showed that exosomal circPAK1 was upregulated in lenvatinib-resistant cells in contrast to parental cells. **G** Intercellular trafficking of exosomes among different cell lines by isolated exosomes labeled with PKH67 dye, scale bar, 100 nm. **H** Treatment with exosomes derived from LM3-LR and Hep-3B-LR cells increased circPAK1level, however this effect was abrogated when LM3-LR and Hep-3B-LR cells were silenced with circPAK1. **I** cell viability in exosome-treated parental cells

To examine whether circPAK1 is involved in mediating HCC lenvatinib resistance, we generated two lenvatinib-resistant HCC cell lines (LM3-LR and Hep-3B-LR). The IC50 of the two lenvatinib-resistant cell lines and their parental cell lines (LM3-P and Hep-3B-P) were shown in Fig. S4F. Next, we performed a qRT-PCR analysis and confirmed that circPAK1 was consistently increased in the two lenvatinib-resistant cell lines than in their parental cell lines (LM3-P and Hep-3B-P) (Fig. 8A). To explore the potential correlations between circPAK1 and lenvatinib resistance in HCC, we first silenced circPAK1 in LM3-LR and Hep-3B-LR cells and confirmed the knockdown efficiency (Fig. 8B). We found that the depletion of circPAK1 remarkably increased the lenvatinib sensitivity of the two lenvatinib-resistant HCC cell lines as determined by cell viability assays (Fig. 8C). Collectively, circPAK1 is critical for maintaining lenvatinib resistance.

It has been reported that circRNAs are abundant in exosomes and can be transferred from cell to cell via exosomes [36–38]. Other reports demonstrated that exosomes could mediate drug resistance by exosomes [12, 13, 39, 40]. Therefore, the subsequent research was mainly focused on whether circPAK1 could confer lenvatinib resistance by integrating into exosomes. We then isolated exosomes from the CM of LM3-LR and Hep-3B-LR cells and their parental cell lines. Exosomes isolated from the CM of LM3-LR and Hep-3B-LR cells were identified by TEM, NTA and WB. The representative micrograph and video were taken by TEM; NTA analysis revealed that the average diameter of exosomes was 100 nm (Fig. 8D). The typical positive exosome biomarkers (Calnexin) and negative exosome biomarkers (Alix, Tsg101 and CD9) were detected by WB (Fig. 8E). In addition, qRT-PCR indicated that exosomes isolated from LM3-LR and Hep-3B-LR CM contained more circPAK1 than those from their parental cell lines, suggesting that exosomes may have the ability to transmit circPAK1 (Fig. 8F).

We used three prolonged stages to explore whether exosome could mediate the transfer of circPAK1 disseminates lenvatinib resistance. Firstly, we labeled exosomes isolated from LM3-LR and Hep-3B-LR cells with PKH67 followed by incubation with their parental cells, respectively, for 48 h. As shown in Fig. 8G, a strong green signal was observed in LM3-P and Hep-3B-P cells, indicating that the exosomes were taken up by recipient parental cells. Next, we determined the level of circPAK1 in recipient parental cells and found that after incubation with exosomes isolated from the lenvatinib-resistant cells, higher levels of circPAK1 were determined, but exosomes from circPAK1 knockdown lenvatinib-resistant cells failed to raise the level of circPAK1 in recipient cells (Fig. 8H). The results of these two stages indicated that exosomes could transport circPAK1 from lenvatinib-resistant cell lines to their parental cell lines. Lastly, we explored whether exosome-transferred circPAK1 could induce the resistance of recipient cells to lenvatinib. Surprisingly, when we treated the parental cells with exosomes isolated from their lenvatinib-resistant cell lines, their sensitivity to lenvatinib was significantly decreased (Fig. 8I). Taken together, our data demonstrated that circPAK1 could be transported by exosomes from lenvatinib-resistant HCC cells to recipient parental cells and confer the resistant phenotype to recipient cells.

## Discussion

HCC is still one of the most lethal malignant tumors, which seriously influences the quality of human life [41]. With the improvement of diagnosis and treatment options, the 5-year survival rate of postoperative HCC increased to a certain extent. Surgical intervention is still the primary treatment option for HCC. However, for radical resection is only suitable for HCC at an early stage, while the early symptoms of HCC are not typical and patients are often diagnosed at an advanced stage, so the treatment options are limited and the therapeutic effects are unoptimistic.

Molecular-targeted therapies are to design the corresponding therapeutic drugs aiming at the established carcinogenic sites (which can be a protein or a gene fragment inside the tumor cells). Despite the remaining molecular-targeted drugs for HCC have gained a lot benefits, drug resistance is still the main reason that affects the efficiency of chemotherapeutic drugs [42, 43]. Therefore, there is an urgent need to identify other effective molecular therapy targets.

Accumulating studies prove that circRNAs play a crucial role in the progression of various malignancies [4–7]. The biological functions of circRNAs are diverse, mainly including serving as miRNA sponge, translating, modulating alternative splicing and transcription, interacting with RNA binding proteins (RBPs), translocating, etc.

In this study, we performed high-throughput sequencing and combined with a series of filter conditions, a significantly upregulated circRNA, circPAK1, was finally identified. Clinically, circPAK1 was significantly upregulated in HCC tissues, and high expression of circPAK1 negatively correlates with tumor size, LN metastasis, TNM stage and MVI. Functionally, a series of in vitro and in vivo experiments demonstrate that circPAK1 could promote the proliferation, invasion and metastasis of HCC. We also noticed that overexpression of circPAK1 leads to apoptosis inhibition and angiogenesis of HCC.

Gene-targeted therapy provides a promising option for treating malignancies that are insensitive to chemotherapy. Nanomedicines encompass various nanocarriers with sizes ranging between 10 nm and 1000 nm. They are highly useful owing to their small size and particle dimension, high aspect ratio, encapsulation ability, and the opportunity to functionalize the surface to deliver loaded cargos, including plasmid DNA, small interfering RNA and mRNA [44–46]. Herein, we generated CS/si-circPAK1 nanocomplexes with Chitosan material to simulate gene-targeted therapy. Surprisingly, by subcutaneous xenograft and lung metastasis models, we found that the application of CS/si-circPAK1 nanocomplexes could inhibit the growth and metastasis of HCC effectively, even better than animal grade si-circPAK1. These findings may bring us inspiration in the construction of HCC circPAK1-targeted drugs.

To clarify what mechanism circPAK1 exercised in HCC progression, we then performed RNA-seq and found that Hippo signaling pathway was highly correlated with the downstream of circPAK1. The hippo pathway is an important signaling transduction pathway in the development of organisms, especially in regulating organ size and inhibiting tumorigenesis and immune response. An indispensable function of the Hippo pathway inhibits the activity of YAP, a putative oncogene whose activity is regulated by phosphorylation and subcellular localization. When the Hippo pathway is activated, YAP is phosphorylated by LATS1/2 kinase and isolated in cytoplasm by binding to 14–3-3 protein, thus leading to the inactivation of YAP [31]. In contrast, the inactivation of Hippo pathway will cause the none-phosphorylated YAP translocate to the nucleus, accordingly interacting with various transcription factors, including members of the transcriptional enhancer factor (TEF) family, which is also called the TEA domain (TEAD) family (TEAD1–4) [30, 47]. TEAD family proteins are widely expressed in tissues, and the YAP/TEAD complex is vital in regulating the expression of genes related to cell proliferation and apoptosis [47]. Our results demonstrate that circPAK1 has a significant regulatory effect on the nucleus translocation of YAP; meanwhile, the downstream effectors of YAP, CTGF and CYR61, were downregulated after the knockdown of circPAK1, but upregulated when circPAK1 overexpression.

Based on the previous study of circRNA in delivering YAP into the nucleus by binding to YAP directly to promote the EMT of CRC [32], we performed RIP to validate whether circPAK1 has similar biological effects. However, the RIP assay failed to confirm this interaction, this made us speculate that there may exist other RBPs mediate the YAP nucleus translocation indirectly. To validate this speculation, we designed the specific probe of circPAK1 and performed the RNA pulldown assay to determine the RBPs of circPAK1. Although the key molecules in the hippo pathway are not involved among these pull-down proteins, it is worth noting that 14–3-3ζ protein showed a strong binding ability with circPAK1. We further confirmed the existence of this binding by RIP and WB. 14–3-3 family proteins play a key regulatory role in signal transduction, checkpoint control, apoptosis and nutrition sensing channels [48, 49]. The binding of 14–3-3 will shade the special sequence of the target protein and then affect the localization phosphorylation state, stability and intermolecular interaction of the target protein [48–51]. The dysregulation of 14–3-3 is highly correlated with the occurrence and development of tumor [52]. At least seven subtypes of 14–3-3, β, γ, ε, σ, ζ, τ and η, have been found in mammals. The spatio-temporal expression patterns of different 14–3-3 protein subtypes were found during the growing development and acute response to extracellular signals and drugs, indicating that although the sequences of 14–3-3 protein subtypes are similar, they may have different functions [51]. Our results showed that circPAK1 competitively binds to 14–3-3ζ with YAP，thus impairing the recruitment and cytoplasm fixation of 14–3-3ζto YAP, consequently promoting the nucleus transportation and the amplification of downstream target genes of YAP.

As a multikinase inhibitor, lenvatinib was approved by the US Food and Drug Administration (FDA) for unresectable HCC patients in 2018. However, drug resistance is still the major hurdle that limits the application and efficiency of Lenvatinib [43]. Therefore, it is extremely important to elucidate the detailed mechanisms for chemoresistance. Multiple lines of evidence have suggested that circRNAs play a pivotal role in regulating drug resistance. For example, Xu et al. [13] reported that they found circRNA, which they named circSORE, could induce sorafenib resistance to HCC. Zhang et al. [53] demonstrate that circMED27 promotes HCC resistance to lenvatinib. Liu et al. [54] revealed that ectopic expression of circKCNN2 in HCC cells enhanced the therapeutic effect of lenvatinib. These results suggest that circRNAs are involved in the regulation of chemoresistance. Since the RNA-seq results showed that the VEGF pathway is also associated with circPAK1, and circPAK1 has been shown to promote angiogenesis in our functional experiments, we speculate that circPAK1 may affect the chemosensitivity of HCC. Surprisingly, circPAK1 was overexpressed in lenvatinib resistance cell lines than their parental cells, and the knockdown of circPAK1 increased the lenvatinib sensitivity of lenvatinib resistance cells, indicating that circPAK1 is crucial for maintaining lenvatinib resistance.

Additionally, we demonstrated a novel mechanism that exosomes released by lenvatinib resistant cells could mediate circPAK1 transfer and transmission of lenvatinib resistance, and this finding is consistent with the recently reported studies on the transmission of chemotherapy resistance by tumor-derived exosomal circRNAs [12, 13, 55]. Our findings may make exosomal circPAK1 a promising biomarker in liquid biopsy for the early identification of lenvatinib resistance and provide new ideas for overcoming lenvatinib resistance.

## Conclusions

In summary, our data indicate that circPAK1 mediates the nucleus localization of YAP by competitively binding 14–3-3 ζ with YAP, leading to the progression of HCC through shutting down the Hippo signaling pathway. The design of CS/si-circPAK1 nanocomplexes showed satisfactory effects in inhibiting the in vivo growth and metastasis of HCC. We also report a new mechanism by which HCC-derived exosomes deliver circPAK1 and promote the transmission of lenvatinib resistance among HCC cells. Taken together, circPAK1 could be a potential therapeutic target for HCC patients and develop efficient strategies to reverse lenvatinib resistance.

## Supplementary Information

**Additional file 1: Fig. S1.** (A) Relative expression of the 5 candidate circRNAs in human HCC tissues and adjacent nontumor tissues of 20 patients. (B) Cell cycle was performed in the three groups. Data were shown as mean ± SEM. **Fig. S2.** (A) Size distribution of CS/si-circPAK1 nanocomplexes (CS/si-circPAK1 = 50/1). (B) Zeta potential of CS/si-circPAK1 nanocomplexes (CS/si-circPAK1 = 50/1). (C) Release curve of si-circPAK1. (D) IHC analysis of Ki-67 in the tumors derived from mice, scale bar, 50 μm. **Fig. S3.** The positive effect of circPAK1 on HCC progression was rescued by YAP silencing. (A) The silencing efficiency of si-YAP in Lv-circPAK1 Hep-3B cells was determined by qRT-PCR. (B) The colony formation assay of Hep-3B cells transfected Lv-circPAK1 and YAP siRNA. (C) EdU incorporation assay of Hep-3B cells transfected Lv-circPAK1 and YAP siRNA, scale bar, 50 μm. (D) Transwell assay assay of Hep-3B cells transfected Lv-circPAK1 and YAP siRNA, scale bar, 200 μm. (E) Wound healing assay of Hep-3B cells transfected Lv-circPAK1 and YAP siRNA, scale bar, 100 μm. (F) HUVEC tube formation, migration and invasion assay of Hep-3B cells transfected Lv-circPAK1 and YAP siRNA, scale bar, 100 μm. *p < 0.05; **p < 0.01; ***p < 0.001. Data were shown as mean ± SEM. **Fig. S4.** (A) RIP-qPCR was performed to determine the association between circPAK1 and AGO2. (B) liquid chromatography mass spectrometry identified the 14–3-3ζ protein. (C) The silencing efficiency of si-14-3-3ζ in sh-circPAK1 LM3 cells was determined by qRT-PCR. (D) CircPAK1 stable overexpression Hep-3B cells were subjected to IP using 14–3-3ζ antibody or control IgG, followed by IB with 14–3-3ζ, p-LATS1/2 and YAP antibody, to confirm and determine the 14–3-3ζ- p-LATS-YAP protein complex. (E) IF assay in stable circPAK1-knockdown and 14–3-3ζsiRNA LM3 cells, scale bar, 20 μm. (F) The IC50 of lenvatinib resistance cell lines and their parental cells. **Table S1.** ShRNA and siRNA sequence used in this study. **Table S2.** Full sequence information of circPAK1. **Table S3.** Primer sequences used in this study. **Table S4.** Antibodies used in this study. **Table S5.** Probes used in this study. **Table S6.** The 44 upregulated circRNAs.

## Data Availability

All data generated or analyzed during this study are included in this published article [and its supplementary information files].

## Abbreviations

CircRNA: Circular RNA
HCC: Hepatocellular carcinoma
WB: Western blot
CS: Chitosan
RIP: RNA immunoprecipitation assay
Co-IP: Co-Immunoprecipitation
DEGs: Differentially expressed genes
TCGA: The Cancer Genome Atlas
FISH: Fluorescence in situ hybridization
LN: Lymph node
MVI: Microvascular invasion
CM: Conditioned medium
HUVEC: Human umbilical vein endothelial cell
RNA-seq: RNA-sequence
YAP: Yes-associated protein
LR: Lenvatinib resistance
VEGF: Vascular endothelial growth factor
