# Supplementary material for: CircPAK1 promotes the progression of hepatocellular carcinoma via modulation of YAP nucleus localization by interacting with 14-3-3ζ
Source: J Exp Clin Cancer Res. 2022 Sep 22;41:281. doi: 10.1186/s13046-022-02494-z (PMC9494907; doi:10.1186/s13046-022-02494-z)
Supplement: Supplementary file 1 — Additional file 1: Fig. S1. (A) Relative expression of the 5 candidate circRNAs in human HCC tissues and adjacent nontumor tissues of 20 patients. (B) Cell cycle was performed in the three groups. Data were shown as mean ± SEM. Fig. S2. (A) Size distribution of CS/si-circPAK1 nanocomplexes (CS/si-circPAK1 = 50/1). (B) Zeta potential of CS/si-circPAK1 nanocomplexes (CS/si-circPAK1 = 50/1). (C) Release curve of si-circPAK1. (D) IHC analysis of Ki-67 in the tumors derived from mice, scale bar, 50 μm. Fig. S3. The positive effect of circPAK1 on HCC progression was rescued by YAP silencing. (A) The silencing efficiency of si-YAP in Lv-circPAK1 Hep-3B cells was determined by qRT-PCR. (B) The colony formation assay of Hep-3B cells transfected Lv-circPAK1 and YAP siRNA. (C) EdU incorporation assay of Hep-3B cells transfected Lv-circPAK1 and YAP siRNA, scale bar, 50 μm. (D) Transwell assay assay of Hep-3B cells transfected Lv-circPAK1 and YAP siRNA, scale bar, 200 μm. (E) Wound healing assay of Hep-3B cells transfected Lv-circPAK1 and YAP siRNA, scale bar, 100 μm. (F) HUVEC tube formation, migration and invasion assay of Hep-3B cells transfected Lv-circPAK1 and YAP siRNA, scale bar, 100 μm. *p < 0.05; **p < 0.01; ***p < 0.001. Data were shown as mean ± SEM. Fig. S4. (A) RIP-qPCR was performed to determine the association between circPAK1 and AGO2. (B) liquid chromatography mass spectrometry identified the 14–3-3ζ protein. (C) The silencing efficiency of si-14-3-3ζ in sh-circPAK1 LM3 cells was determined by qRT-PCR. (D) CircPAK1 stable overexpression Hep-3B cells were subjected to IP using 14–3-3ζ antibody or control IgG, followed by IB with 14–3-3ζ, p-LATS1/2 and YAP antibody, to confirm and determine the 14–3-3ζ- p-LATS-YAP protein complex. (E) IF assay in stable circPAK1-knockdown and 14–3-3ζsiRNA LM3 cells, scale bar, 20 μm. (F) The IC50 of lenvatinib resistance cell lines and their parental cells. Table S1. ShRNA and siRNA sequence used in this study. Table S2. Full sequ [file 13046_2022_2494_MOESM1_ESM.docx]

**Supplementary information**

**Figure S1.**

(A) Relative expression of the 5 candidate circRNAs in human HCC tissues and adjacent nontumor tissues of 20 patients.

(B) Cell cycle was performed in the three groups. Data were shown as mean ± SEM.


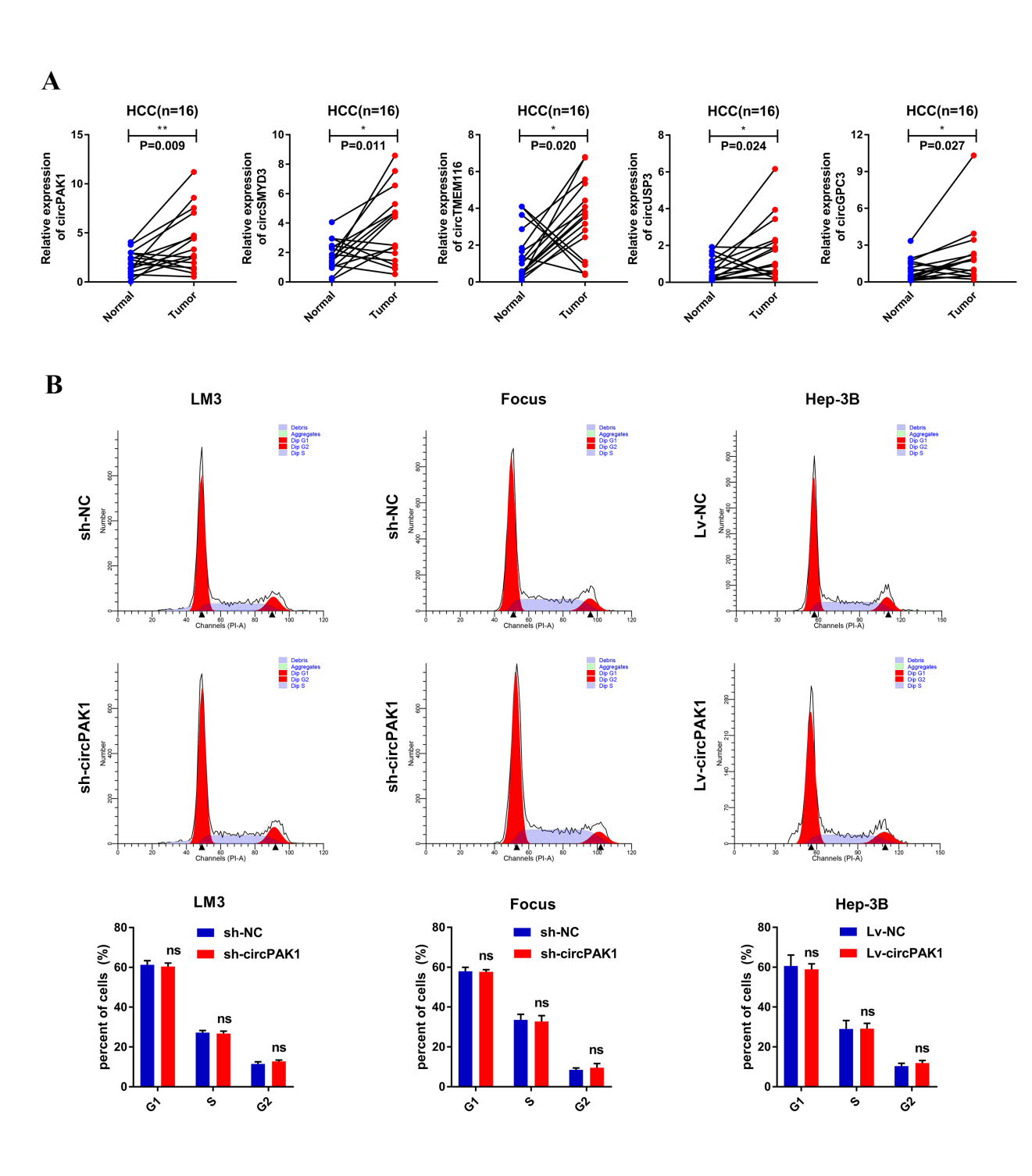


**Figure S2.**

(A) Size distribution of CS/si-circPAK1 nanocomplexes (CS/si-circPAK1=50/1).

(B) Zeta potential of CS/si-circPAK1 nanocomplexes (CS/si-circPAK1=50/1).

(C) Release curve of si-circPAK1.

(D) IHC analysis of Ki-67 in the tumors derived from mice, scale bar, 50μm.


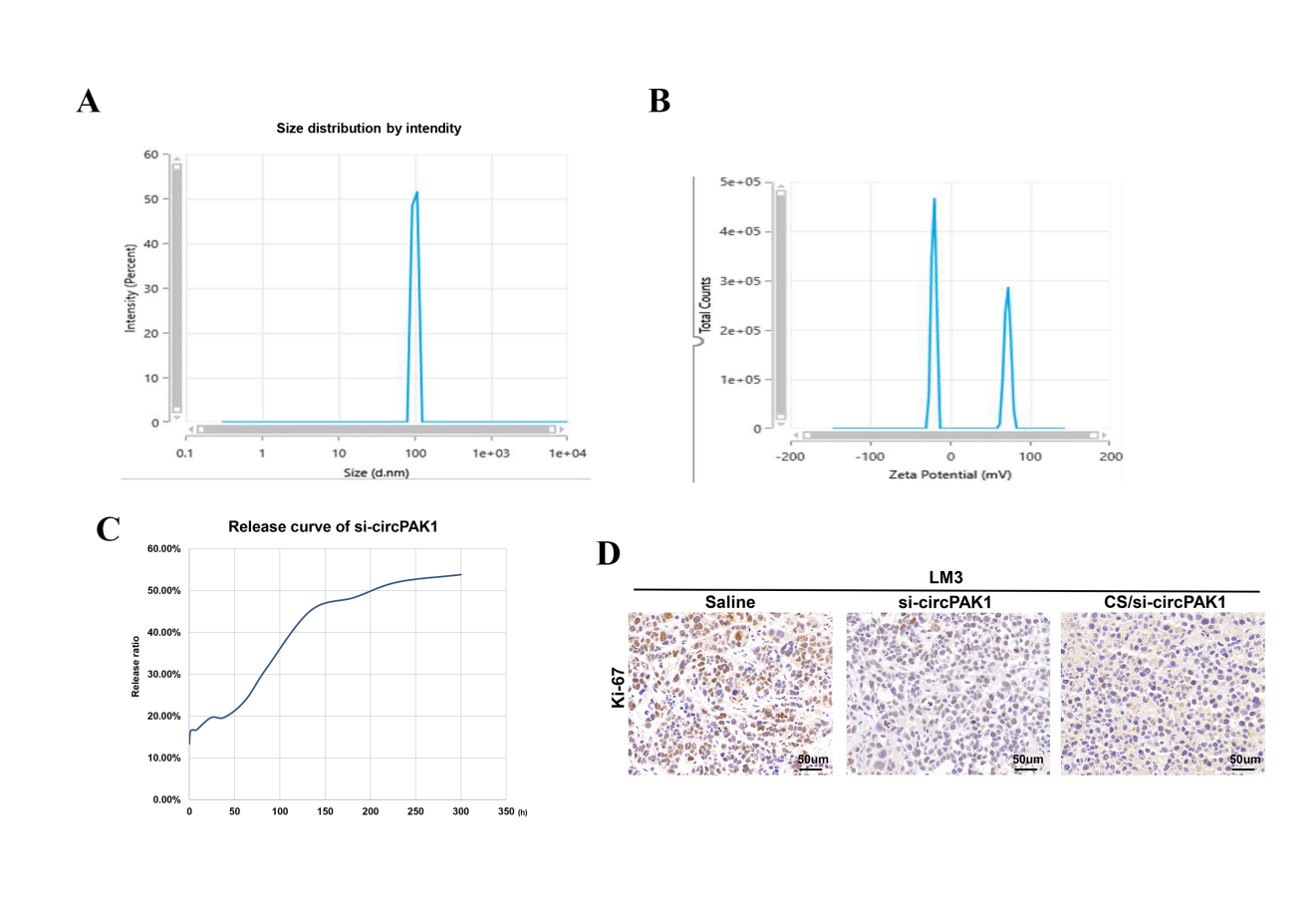


**Figure S3. The positive effect of circPAK1 on HCC progression was rescued by YAP silencing.**

(A) The silencing efficiency of si-YAP in Lv-circPAK1 Hep-3B cells was determined by qRT-PCR.

(B) The colony formation assay of Hep-3B cells transfected Lv-circPAK1 and YAP siRNA.

(C) EdU incorporation assay of Hep-3B cells transfected Lv-circPAK1 and YAP siRNA, scale bar, 50μm.

(D) Transwell assay assay of Hep-3B cells transfected Lv-circPAK1 and YAP siRNA, scale bar, 200μm.

(E) Wound healing assay of Hep-3B cells transfected Lv-circPAK1 and YAP siRNA, scale bar, 100μm.

(F) HUVEC tube formation, migration and invasion assay of Hep-3B cells transfected Lv-circPAK1 and YAP siRNA, scale bar, 100μm. *p < 0.05; **p < 0.01; ***p < 0.001. Data were shown as mean ± SEM.


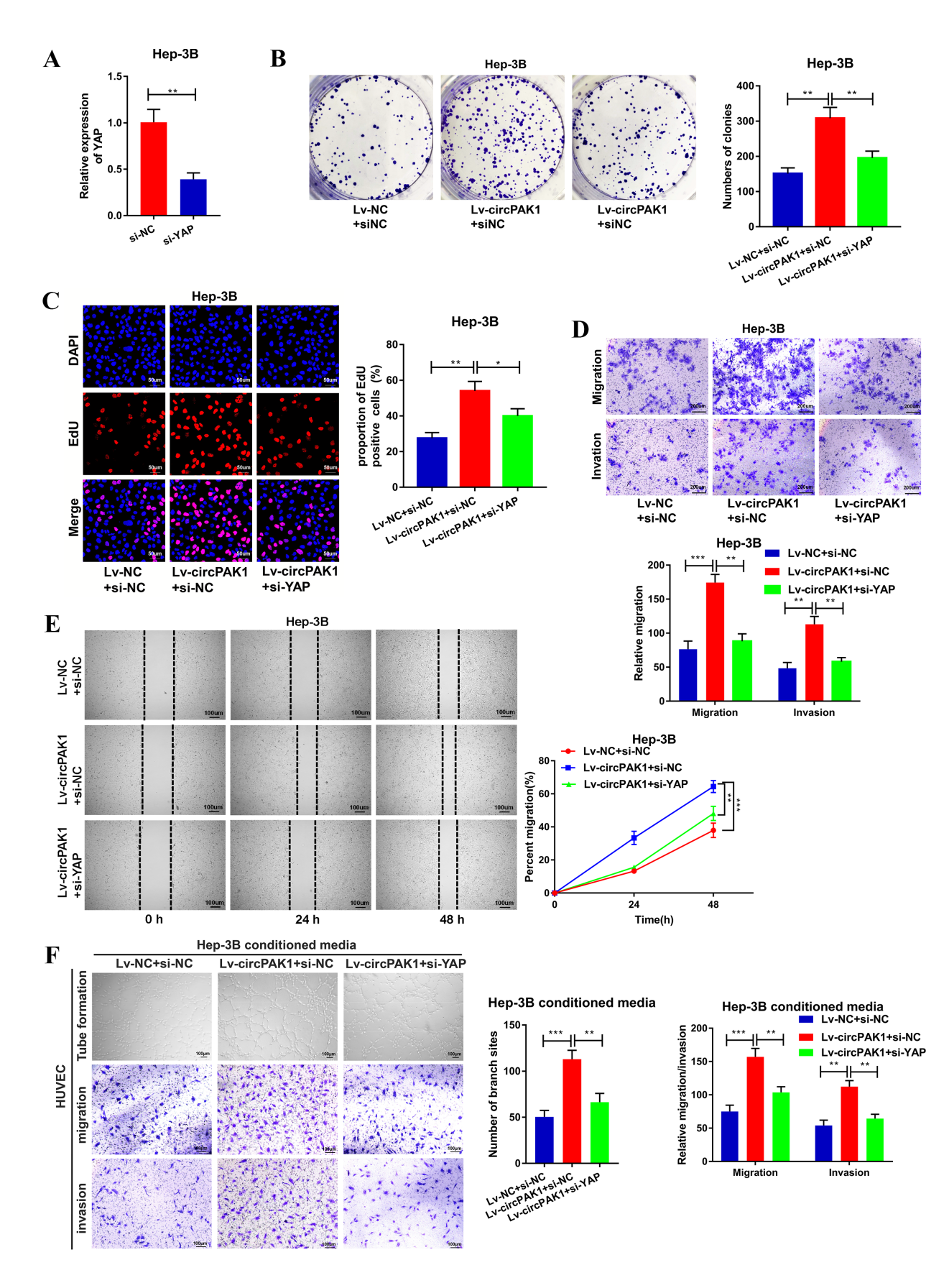


**Figure S4.**

(A) RIP-qPCR was performed to determine the association between circPAK1 and AGO2.

(B) liquid chromatography mass spectrometry identified the 14-3-3ζ protein.

(C) The silencing efficiency of si-14-3-3ζ in sh-circPAK1 LM3 cells was determined by qRT-PCR.

(D) CircPAK1 stable overexpression Hep-3B cells were subjected to IP using 14-3-3ζ antibody or control IgG, followed by IB with 14-3-3ζ, p-LATS1/2 and YAP antibody, to confirm and determine the 14-3-3ζ- p-LATS-YAP protein complex.

(E) IF assay in stable circPAK1-knockdown and 14-3-3ζsiRNA LM3 cells, scale bar, 20μm.

(F) The IC50 of lenvatinib resistance cell lines and their parental cells.

**
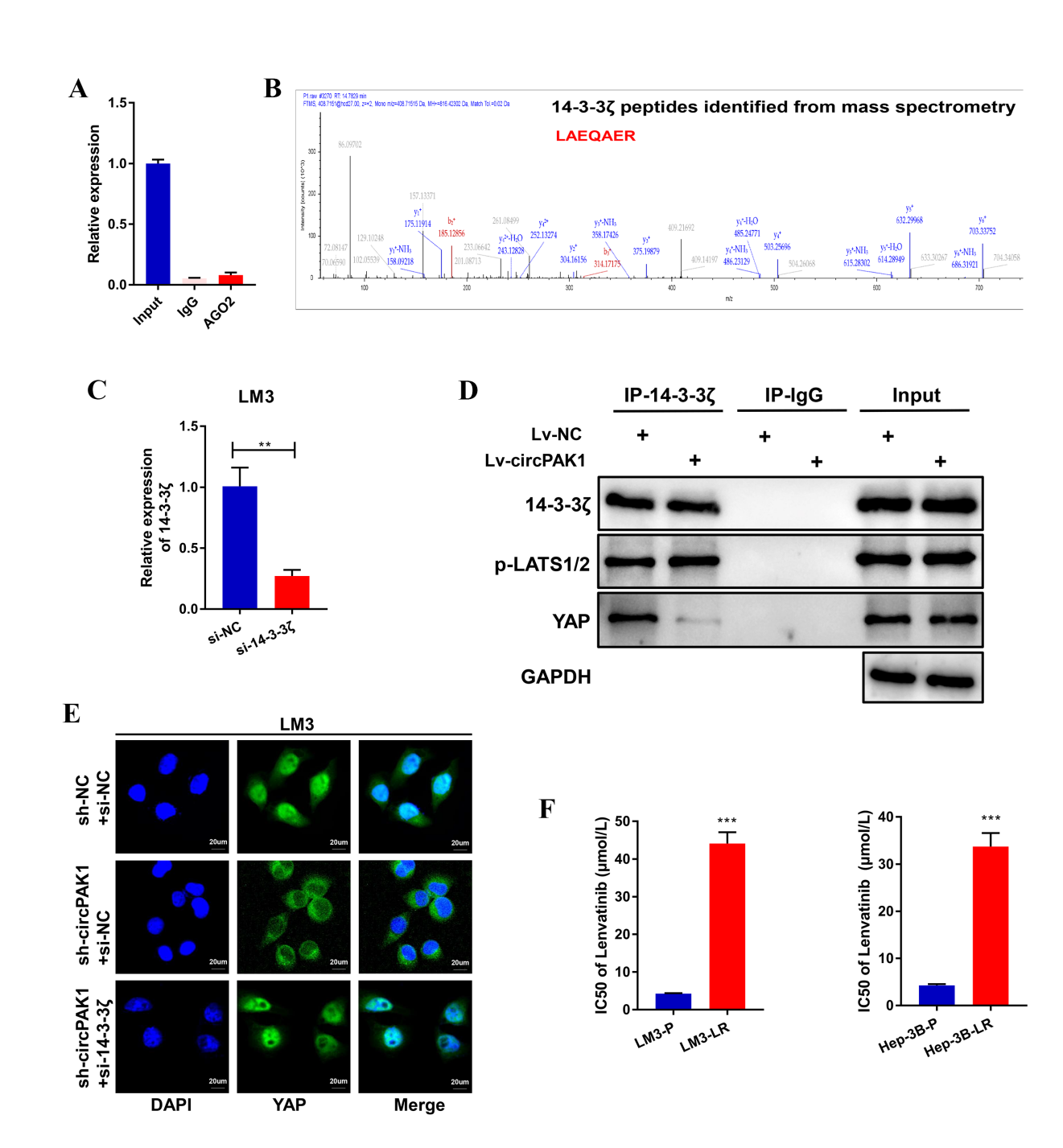
**

**Table S1. ShRNA and siRNA sequence used in this study.**

| sh-RNA and si-RNA | Target sequence (5’-3’) |
| --- | --- |
| sh1-circPAK1 | TTCTAATGCCTTGTAGCTGCT |
| sh-circPAK1-NC | gatctgatagcttcttcgtttttt |
| si-14-3-3ζ | AGCCTGCATGAAGTCTGTAACTGAGCAAG |
| si-14-3-3ζ-NC | CACAAGCTGGAGTACAACTACAACAGCCA |
| si-YAP | CAGTGGCACCTATCACTCT |
| si-YAP -NC | aattaaaaaacgaagaagctatca |

**Table S2. Full sequence information of circPAK1.**

| >hsa_circ_0023685 |
| --- |
| TAGCTGCTGCTGGTGGTGACAATGTCAAATAACGGCCTAGACATTCAAGACAAACCCCCAGCCCCTCCGATGAGAAATACCAGCACTATGATTGGAGCCGGCAGCAAAGATGCTGGAACCCTAAACCATGGTTCTAAACCTCTGCCTCCAAACCCAGAGGAGAAGAAAAAGAAGGACCGATTTTACCGATCCATTTTACCTGGAGATAAAACAAATAAAAAGAAAGAGAAAGAGCGGCCAGAGATTTCTCTCCCTTCAGATTTTGAACACACAATTCATGTCGGTTTTGATGCTGTCACAGGGGAGTTTACGGGAATGCCAGAGCAGTGGGCCCGCTTGCTTCAGACATCAAATATCACTAAGTCGGAGCAGAAGAAAAACCCGCAGGCTGTTCTGGATGTGTTGGAGTTTTACAACTCGAAGAAGACATCCAACAGCCAGAAATACATGAGCTTTACAGATAAGTCAGCTGAGGATTACAATTCTTCTAATGCCTTG |

**Table S3. Primer sequences used in this study.**

| Gene | | Sequence (5’-3’) |
| --- | --- | --- |
| PAK1 | F | CAGCCCCTCCGATGAGAAATA |
|  | R | CAAAACCGACATGAATTGTGTGT |
| circPAK1-divergent | F | TTCTTCTAATGCCTTGTAGC |
|  | R | GTTTAGGGTTCCAGCATCTT |
| circPAK1-convergent | F | AAGAGCGGCCAGAGATTTCT |
|  | R | TCTGGCTGTTGGATGTCTTCT |
| GAPDH | F | GGAGCGAGATCCCTCCAAAAT |
|  | R | GGCTGTTGTCATACTTCTCATGG |
| GAPDH-divergent | F | GAAGACTGTGGATGGCCCCT |
|  | R | CAAATGAGCCCCAGCCTTCT |
| YAP | F | TAGCCCTGCGTAGCCAGTTA |
|  | R | TCATGCTTAGTCCACTGTCTGT |
| 14-3-3ζ | F | ACCAGTATGTAGGCAGTTTTC |
|  | R | GGATCCATGGATAAAGAGCTGGTT |
| Human-CTGF | F | ATCTTCGGTGGTACGGTGT |
|  | R | GTGTCTTCCAGTCGGTAAGC |
| Human-Cy61 | F | GCTTGGCGCAGACCTTACAGCA |
|  | R | TTGACCAGGCTGGCGCTCTC |

**Table S4. Antibodies used in this study**

| Antibody | Source |
| --- | --- |
| Anti-AGO2 antibody | Cell Signaling Technology |
| Anti-14-3-3ζantibody | Cell Signaling Technology |
| Anti-YAP antibody | Abmart |
| Anti-p-YAP (ser27) antibody | Abmart, |
| Anti-LATS1/2 antibody | Abmart |
| Anti-p- LATS1/2  (ser909, ser872) antibody | Abmart |
| Anti-GAPDH antibody | Abcam |
| Secondary antibody | Abcam |
| Anti-Ki67 antibody | Abcam |
| Anti-IgG antibody | Cell Signaling Technology |

**Table S5. Probes used in this study**

| Gene | Probe sequence (5’-3’) |
| --- | --- |
| circPAK1 _(Fish)_ | AGCGATGTGCATTTTGTAAGC |
| circPAK1 _(Pull down)_ | AGCAGCTACAAGGCATTAGA |
| NC _(Pull down)_ | GAATGCAAGTCAATAGCAGA |

**Table S6. The 44 upregulated circRNAs**

| GeneID | circBase ID | Symbol | Named by us  (circ+symbol) |
| --- | --- | --- | --- |
| chrX:132887509\|132888203 | hsa_circ_0091581 | GPC3 | circGPC3 |
| chr13:64576517\|64608670 | hsa_circ_0030389 | TCONS |  |
| chr6:170632167\|170657411 | hsa_circ_0078773 | FAM120B |  |
| chr8:98817581\|98837381 | hsa_circ_0003214 | LAPTM4B |  |
| chr18:12999420\|13001581 | hsa_circ_0046995 | CEP192 |  |
| chr10:96005703\|96006378 | hsa_circ_0019225 | PLCE1 |  |
| chr19:55610152\|55614936 | hsa_circ_0000958 | PPP1R12C |  |
| chr19:35510095\|35510437 | hsa_circ_0005731 | GRAMD1A |  |
| chr1:246021798\|246027188 | hsa_circ_0017286 | SMYD3 | circSMYD3 |
| chr22:42912017\|42973104 | hsa_circ_0001242 | RRP7A |  |
| chr12:112370390\|112381173 | hsa_circ_0028319 | TMEM116 | circTMEM116 |
| chr4:7870349\|7873807 | hsa_circ_0069152 | AFAP1 |  |
| chr1:176050288\|176105683 | hsa_circ_0003572 | RFWD2 |  |
| chr14:91409422\|91413894 | hsa_circ_0032939 | RPS6KA5 |  |
| chr5:14270934\|14287172 | hsa_circ_0005323 | TRIO |  |
| chr1:246490503\|246518396 | hsa_circ_0005034 | SMYD3 |  |
| chr10:124151819\|124159904 | hsa_circ_0020262 | PLEKHA1 |  |
| chr1:246091233\|246093239 | hsa_circ_0017304 | SMYD3 |  |
| chr1:155686797\|155695810 | hsa_circ_0014611 | DAP3 |  |
| chr1:150429764\|150430046 | hsa_circ_0014014 | RPRD2 |  |
| chr5:96314842\|96322374 | hsa_circ_0001513 | LNPEP |  |
| chr15:65110145\|65111422 | hsa_circ_0002205 | PIF1 |  |
| chr11:46811626\|46812133 | hsa_circ_0007985 | CKAP5 |  |
| chr20:48256215\|48257202 | hsa_circ_0004868 | B4GALT5 |  |
| chr15:63824846\|63855207 | hsa_circ_0002138 | USP3 | circUSP3 |
| chr3:9792629\|9796569 | hsa_circ_0064155 | OGG1 |  |
| chr1:235993526\|235996967 | hsa_circ_0005899 | LYST |  |
| chr21:27462259\|27484463 | hsa_circ_0061349 | APP |  |
| chr7:148543562\|148544397 | hsa_circ_0006357 | EZH2 |  |
| chr12:122745808\|122748746 | hsa_circ_0029062 | VPS33A |  |
| chr1:86241357\|86252144 | hsa_circ_0005110 | COL24A1 |  |
| chr2:232952197\|232952431 | hsa_circ_0058691 | DIS3L2 |  |
| chr4:108603171\|108622441 | hsa_circ_0006935 | PAPSS1 |  |
| chr7:44663916\|44687358 | hsa_circ_0080056 | OGDH |  |
| chr1:45223228\|45223812 | hsa_circ_0002563 | KIF2C |  |
| chr4:71628236\|71634378 | hsa_circ_0002048 | RUFY3 |  |
| chr4:2626988\|2632896 | hsa_circ_0004517 | FAM193A |  |
| chr9:96305504\|96312973 | hsa_circ_0087557 | FAM120A |  |
| chr11:3726430\|3727857 | hsa_circ_0020875 | NUP98 |  |
| chr1:230798887\|230800333 | hsa_circ_0016866 | COG2 |  |
| chr17:64092369\|64128881 | hsa_circ_0045355 | CEP112 |  |
| chr1:183083666\|183084772 | hsa_circ_0004573 | LAMC1 |  |
| chr5:167995651\|167996003 | hsa_circ_0006894 | PANK3 |  |
| chr11:77085373\|77103586 | hsa_circ_0023685 | PAK1 | circPAK1 |
